# Causal signals between codon bias, mRNA structure, and the efficiency of translation and elongation

**DOI:** 10.15252/msb.20145524

**Published:** 2014-12-23

**Authors:** Cristina Pop, Silvi Rouskin, Nicholas T Ingolia, Lu Han, Eric M Phizicky, Jonathan S Weissman, Daphne Koller

**Affiliations:** 1 Computer Science Department, Stanford UniversityStanford, CA, USA; 2 Department of Cellular and Molecular Pharmacology, California Institute of Quantitative Biology, Center for RNA Systems Biology, Howard Hughes Medical Institute, University of CaliforniaSan Francisco, CA, USA; 3 Department of Molecular and Cell Biology, University of California, BerkeleyBerkeley, CA, USA; 4 School of Medicine and Dentistry, University of Rochester Medical CenterRochester, NY, USA

**Keywords:** codon usage bias, elongation, mRNA structure, translation efficiency

## Abstract

Ribosome profiling data report on the distribution of translating ribosomes, at steady-state, with codon-level resolution. We present a robust method to extract codon translation rates and protein synthesis rates from these data, and identify causal features associated with elongation and translation efficiency in physiological conditions in yeast. We show that neither elongation rate nor translational efficiency is improved by experimental manipulation of the abundance or body sequence of the rare AGG tRNA. Deletion of three of the four copies of the heavily used ACA tRNA shows a modest efficiency decrease that could be explained by other rate-reducing signals at gene start. This suggests that correlation between codon bias and efficiency arises as selection for codons to utilize translation machinery efficiently in highly translated genes. We also show a correlation between efficiency and RNA structure calculated both computationally and from recent structure probing data, as well as the Kozak initiation motif, which may comprise a mechanism to regulate initiation.

## Introduction

The translation of RNA into protein is the nexus of decoding genetic information into functional polypeptides and also a central biosynthetic process consuming a substantial fraction of the cell's resources. Although apparently redundant nucleotide sequences encode each protein, usage of different synonymous codons is highly biased (Plotkin & Kudla, 2011). These preferences are strongest in highly expressed genes throughout diverse organisms (Man & Pilpel, 2007; Hershberg & Petrov, 2008), suggesting selective pressure for the efficient use of the translational apparatus during the synthesis of abundant proteins. At the same time, less common codons may be used in order to modulate translation or may arise due to competing sequence constraints such as mRNA secondary structure. While the evolutionary signature of codon bias is clear, its biochemical basis remains unsettled.

Ribosome profiling (Ingolia *et al*, 2009) is an emerging technique for profiling translation *in vivo* that is well suited to provide insights into the factors controlling the speed of translation as well as the amounts of each protein produced by the cell. Ribosome profiling data comprise a set of ribosome-protected fragments (*footprints*) marking ribosome density along mRNA transcripts with codon resolution. We can therefore extract from these data both the yield of each protein (*protein synthesis rate*) and the rate at which each codon is translated (*codon translation rate* or *elongation rate*). However, estimation of these two quantities is nontrivial, and *ad hoc* approaches disregard differences in elongation rates between genes or exclude mRNAs with sparse footprint coverage. A number of studies with different analysis approaches present varying hypotheses for the mechanisms underlying variation in elongation and *translation efficiency* in yeast and other organisms (Tuller *et al*, 2010a,b, 2011; Ingolia *et al*, 2011; Stadler & Fire, 2011; Qian *et al*, 2012; Charneski & Hurst, 2013; Shah *et al*, 2013; Woolstenhulme *et al*, 2013; Gardin *et al*, 2014; Lareau *et al*, 2014). These include codon effects mediated by tRNA abundance or wobble base pairing, as well as effects of mRNA structure and the nascent peptide on the ribosome.

Here, we present a rigorous statistical method that estimates, from ribosome profiling data, both elongation rates and protein synthesis levels on individual transcripts; as a byproduct, it also estimates *translation efficiency* (TE), the propensity of a transcript to generate complete protein, defined as the total amount of protein produced from an mRNA message, and calculated here as our model-derived protein synthesis rates divided by the mRNA levels. We use our robust modeling framework in conjunction with new high-resolution data from wild-type yeast, along with three tRNA mutants, to explore some of the conflicting views on the causality between codon usage and elongation rate, as well as between codon usage and TE, in physiological conditions at a genome-wide level.

We first apply our model to examine biological factors contributing to local translation kinetics. Due to differences in tRNA levels that correlate with synonymous codon bias, variability in codon translation rates observed per gene is commonly thought to be governed by the abundance of cognate tRNAs (Varenne *et al*, 1984; Sorensen *et al*, 1989). However, codon bias does not correlate with indirect measures of decoding speed, at least in bacteria (Bonekamp *et al*, 1989; Curran & Yarus, 1989). Similar to other observations in ribosome profiling datasets (Li *et al*, 2012; Qian *et al*, 2012; Charneski & Hurst, 2013), we find that codon usage bias is a poor predictor of elongation rate. We further test for causal influence and illustrate that experimentally manipulating tRNA abundance or body similarly does not affect the elongation rate when decoding with the manipulated tRNA. In addition, our model identifies positions where elongation is slower than expected based on codon identity and suggests that such pauses commonly occur closer to the 5′ end but are unrelated to codon bias.

Finally, we use our model to disentangle the factors underlying message-specific differences in translational efficiency. In physiological conditions, initiation rather than elongation may largely determine overall protein production; initiation predominates when it is slow relative to the time needed to elongate through the width of one ribosome (∼10 codons), so that translating ribosomes rarely interfere with each other, and when elongation is highly processive, so that most initiation events result in a protein (Andersson & Kurland, 1990; Bulmer, 1991; Arava *et al*, 2003; Lackner & Bahler, 2008). Analysis of our tRNA-perturbed mutant experiments shows that efficiency is not causally affected by improving tRNA levels, leading us to focus on initiation signals in understanding variation in translational efficiency across different messages. Several causes for slow initiation have been proposed: codon bias at the 5′ end (Tuller *et al*, 2010a, 2011), secondary structure (Kudla *et al*, 2009; Gu *et al*, 2010; Kertesz *et al*, 2010; Tuller *et al*, 2011; Keller *et al*, 2012; Zur & Tuller, 2012), and gene length (Arava *et al*, 2005; Lackner *et al*, 2007; Ding *et al*, 2012). We find that a Kozak-like initiation motif (Kozak, 1981) and lack of structure around the start codon are predictors of TE. Overall, our experimental and analytical results provide support to a previously proposed model in which initiation is rate-limiting in physiological conditions (Bulmer, 1991), in which initiation rate is affected largely by mRNA sequence features, and where translational efficiency is not significantly affected by codon usage (Andersson & Kurland, 1990; Bulmer, 1991). In contrast with experiments in non-physiological conditions, our results endorse the resulting explanation that, in endogenous conditions, perhaps in combination with other pressures, selection for efficient use of ribosomes and associated factors in the synthesis of highly translated proteins is a potential driver of the observed codon usage biases.

## Results

### Queuing model for elongation process

To extract high-quality estimates of protein synthesis rates and codon translation rates from the ribosome footprint data, we model the process of ribosome flow, using gene- and codon-dependent parameters, and the physical sampling that occurs in the experimental protocol from which these data are derived. Our design choices are motivated by potential biases in the data including sparse footprint counts for low abundance genes, biases due to the position along the mRNA, and biases due to the identity of the mRNA.

**Figure 1:**
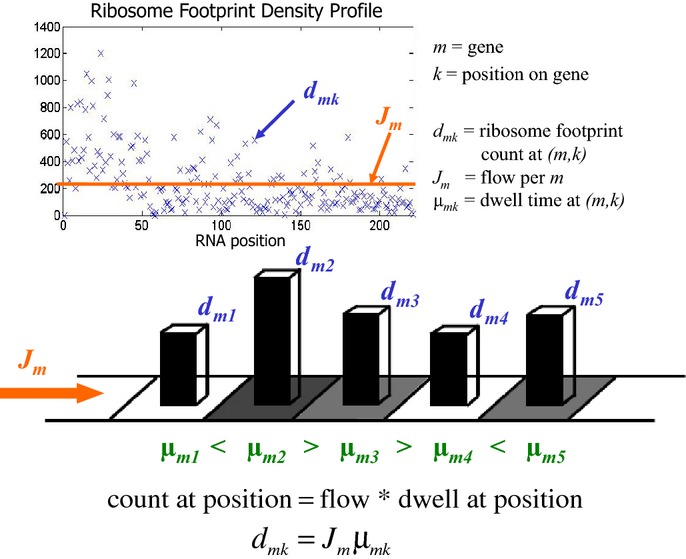
Model of protein synthesis Ribosomes initiate translation with a protein synthesis rate or flow (*J*) of ribosomes. This is conserved across the strand, so that at each residue (*m,k*) the flow depends on the dwell time of the ribosome (μ) and the ribosome occupancy (proportional to footprint count *d*). Slower positions, for example, (*m,2*) compared to (*m,1*), can inflate the average footprint count per gene and must be accounted for when estimating flow. Dwell times and flow are correlated with local and global *cis*-features.

Our model inputs are the set of ribosome footprint counts *d* at each codon in the genome, sparsely sampled (due to sequencing depth) from an unobserved steady-state distribution π. In particular, *d*_*mk*_ is the observed footprint count at position *k* in mRNA message *m* and π_*mk*_ encodes the fraction of ribosomes at (*m,k*). Consequently, the distribution must satisfy *flow conservation constraints*: If ribosomes do not fall off the message, then due to conservation of matter, the protein synthesis rate *J*_*m*_ for message *m* (the ribosome flow out of the stop codon) must be the same as the flow *J*_*mk*_ from any position *k* on *m*. If we define μ_*mk*_ as the dwell time of the ribosome at (*m,k*)*,* flow conservation also implies that rapidly translating positions (small μ_*mk*_) are occupied for a smaller fraction of time (small π_*mk*_) than positions that are slow to translate. The dwell time μ_*mk*_ is the inverse of the rate at which the ribosome elongates off of position (*m,k*) and so intuitively depends on the amount of time the ribosome requires to perform one elongation step (recruit tRNA, form the peptide bond, and translocate). Thus, at steady-state, flow *J*_*mk*_ is proportional (up to a constant encoding the number of ribosomes in the system) to π_*mk*_*/*μ_*mk*_, where we use *d*_*mk*_ throughout as our observed proxy for π_*mk*_. Figure1 shows the relationship between the variables.

We use the counts *d* to estimate the quantities {μ_*mk*_} and {*J*_*m*_} in a novel probabilistic regression accounting for flow conservation and assuming steady-state and no ribosome falloff. Briefly, we optimize over two terms: 

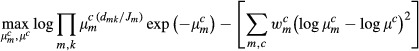


The first term is a standard likelihood term for the data, using a model encoding flow conservation. Since a single ribosome profiling dataset does not contain enough data to robustly infer a separate μ_*mk*_ for each *(m,k)*, we use the same dwell time 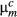 for every occurrence of the same codon *c* within message *m*, making 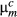 an expected dwell time for codon *c* on message *m*. The second term additionally softly constrains 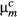 to be similar to a global codon dwell μ^*c*^, based on the intuition that the same codon behaves similarly throughout the cell. To optimize the objective, we (i) estimate the dwell times 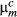 and μ^*c*^ with flow *J*_*m*_ fixed and (ii) set flow *J*_*m*_ to be the average of the flows *J*_*mk*_ (namely the dwell-corrected footprint counts *d*_*mk*_/μ_*mk*_) across each message: *J*_*m*_ = Σ_*k in m*_ (*d*_*mk*_/μ_*mk*_)/*L*_*m*_ (see Materials and Methods for details).

We ran our model on a ribosome profiling dataset gathered for *Saccharomyces cerevisiae* in rich medium, using a flash-freezing technique as described before (Ingolia *et al*, 2012) (see Materials and Methods). To verify the validity of our estimated parameters, we compared our protein synthesis rate *J*_*m*_ to two external measures of protein abundance—GFP-based levels from Newman *et al* (2006) and mass-spectrometry-based levels from De Godoy *et al* (2008)—and obtained strong correlations (Pearson *r* = 0.789 and 0.680, respectively, *P* = 0). These improve on the protein abundance estimates from Ingolia *et al* (2009), computed as the simple average of (uncorrected) footprint counts per message (Supplementary Fig S1). While correlation with these standard estimates of protein abundance is reassuring, these methods have general limitations such as ascertainment bias for less abundant proteins as well as technical limitations such as the impact of fusion tags on protein levels. In addition, ribosome profiling measures translation and protein synthesis, but steady-state protein abundance is also affected by rates of protein degradation.

While the protein synthesis flux is perhaps the most obvious interesting quantity that can be extracted from profiling data, we can also derive other quantities of interest from our learned model parameters. We compute translation efficiency *TE*_*m*_ of a given mRNA molecule *m* by dividing protein synthesis rate *J*_*m*_ by mRNA transcript levels *M*_*m*_, derived from mRNA fragment data collected separately in the ribosome footprinting experiment. We can identify codon-dependent effects on translation from differences in μ^*c*^. By looking at footprint count deviation from expected dwell time at each *(m,k)*, we can also examine differences among codons on the same message. In the following sections, using the parameters estimated under our robust probabilistic framework, we perform a comprehensive analysis of the biological factors influencing local and global dynamics of translation.

### Codon translation is not affected by tRNA abundance or body sequence

**Figure 2:**
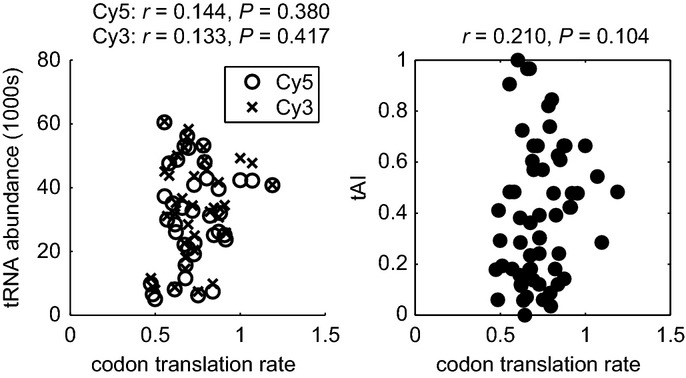
Correlation between codon translation rates and measures of codon usage bias Left: Insignificant Spearman correlation between estimated codon translation rates (scaled up by a factor of 1,000) and tRNA abundance from microarray measurements using either fluorophore Cy3 or Cy5 (Dittmar *et al*, 2004) on 39 codons with measured levels. Right: The same correlation but to tAI is also not significant.

A number of studies in *Escherichia coli* initially identified codon usage and the availability of tRNA as the dominant force for codon translation rate (Varenne *et al*, 1984; Sorensen *et al*, 1989). Later studies found no correlation between measured rates and tRNA abundance or codon frequency (Bonekamp *et al*, 1989; Curran & Yarus, 1989; Sorensen & Pedersen, 1991). However, all of these studies measured translation speed indirectly, on individual and potentially idiosyncratic reporter systems. We explore these competing hypotheses in the physiological conditions of our yeast dataset. If tRNA abundance were rate-limiting for elongation, we would expect a positive correlation between codon translation rate and tRNA abundance. However, as shown in Fig2, the correlation is insignificant (Spearman *r* = 0.144, *P* = 0.380 for Cy5 and *r* = 0.133, *P* = 0.417 for Cy3 from microarray tRNA measurements (Dittmar *et al*, 2004)). A similar result (*r* = 0.210, *P* = 0.104) is also obtained when comparing to tAI, a measure of codon bias based on tRNA gene copy number relative to the overall collection of isoacceptor tRNAs (Dos Reis *et al*, 2004). If we restrict the analysis to the slowest synonymous codon (in terms of tAI), to the fastest, or to the average per amino acid, the correlation with tAI does not improve: *r* = −0.12 (*P* = 0.61), *r* = −0.29 (*P* = 0.22), and *r* = −0.32 (*P* = 0.18), respectively. Finally, the same insignificant correlation exists in the raw footprint data (*r* = 0.112, *P* = 0.392; baseline method for rate described in Materials and Methods) and was also observed in another analysis of the yeast dataset from Ingolia *et al* (2009), in which codon dwell time was estimated as the ratio of observed codon frequencies in the footprint data relative to expected codon frequencies in the mRNA fragment data (Qian *et al*, 2012).

**Figure 3:**
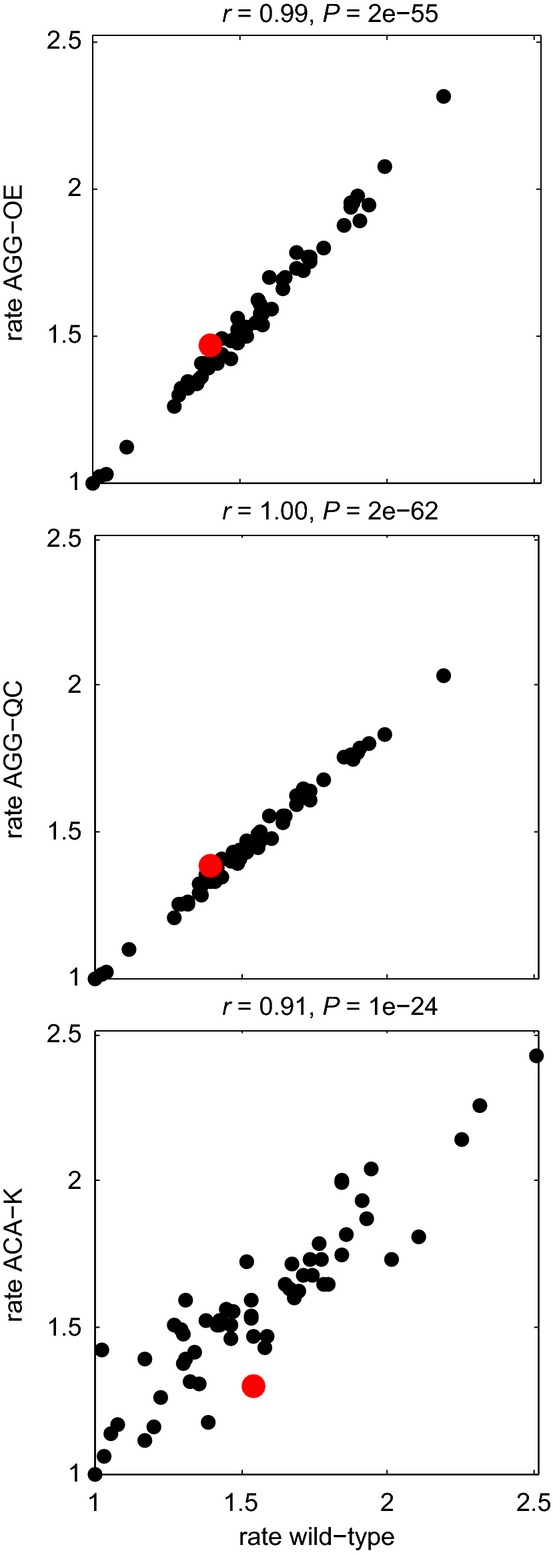
Comparison between codon translation rates in wild-type and mutants Correlation between estimated codon translation rates in wild-type versus mutant for the three mutant samples (the manipulated codon is highlighted in red). Rates are normalized by the minimum one in each sample. Pearson correlations are nearly exact, indicating that the mutant rates are generally unaffected.Source data are available online for this figure.

Our analysis of elongation rates on endogenous mRNAs in the context of the co-adapted cellular tRNA pool addresses the effects of codon usage in natural physiology, but may be confounded by this co-adaptation and cannot directly test the causal links between various correlated mRNA features. To measure the effect of tRNA abundance on codon translation rate directly, we created three mutant yeast species to test whether (i) tRNA overexpression speeds up translation, (ii) the tRNA body itself causes the tRNA-dependent rate effect observed in other studies, or (iii) depletion of tRNA slows down ribosomes. In our first mutant, AGG-OE, the tRNA recognizing AGG (namely tRNA^Arg(CCU)^) was overexpressed on a high-copy plasmid; in mutant AGG-QC, the body sequence of the tRNA recognizing AGG was swapped with the body of a more preferred tRNA (as measured by tAI); and in mutant ACA-K, three out of four copies of the tRNA recognizing ACA were deleted from the genome. The AGG mutants had a URA marker and were compared against a wild-type sample with a URA plasmid (see Materials and Methods). For ACA-K, we checked that the abundance of the tRNA for ACA (namely tRNA^Thr(UGU)^) did decrease to about 30% of wild-type (Supplementary Table S1). In the AGG-OE mutant, we measured the amount of total and aminoacylated tRNA for tRNA^Arg(CCU)^ (see Materials and Methods) and verified that the tRNA was overexpressed by 13.8-fold (±0.4), based on an analysis of two independently derived RNA samples, and remained charged at a level similar to wild-type (87%) (Supplementary Fig S2). For the AGG-QC mutant, we similarly verified that the amount of charged tRNA^Arg(CCU)^ was similar to wild-type (Supplementary Fig S2). We generated ribosome profiling data and ran our model on these mutants to test whether AGG codons are translated faster in AGG-OE and AGG-QC and whether ACA codons are translated slower in ACA-K. We observe no significant change in the elongation rates of the affected codon in any of the three mutants compared to wild-type (Fig3, Supplementary Fig S3); the overall correlation between ACA-K and wild-type is not as tight as for other mutants, but this is due to changes affecting all codons, not only ACA. We verified the result by inspecting the footprint counts at the perturbed codon relative to adjacent counts in the mutants compared to wild-type and saw no unusual increase or decrease (Supplementary Fig S4). One prevailing hypothesis (Welch *et al*, 2009) is that the amount of charged as opposed to total tRNA is the true predictor of codon elongation; our measurements of aminoacylated tRNA suggest that these levels were manipulated as expected and that this is not a confounding factor in the mutant samples. Hence, our results suggest that several-fold changes in tRNA abundance do not affect ribosome dwell time.

### Translation efficiency is mildly affected by tRNA knockdown but not by overexpression

**Figure 4:**
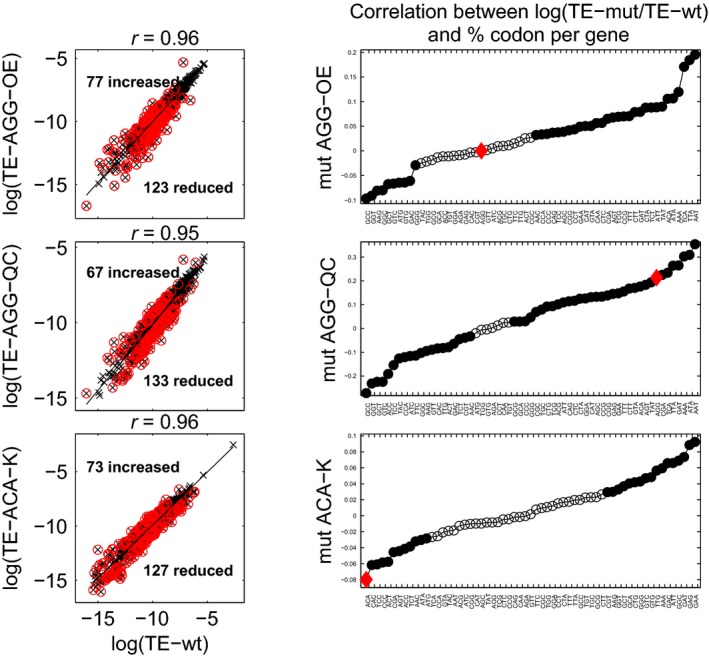
Comparison between translation efficiency in wild-type and mutants Left: Wild-type TE compared to mutant TE for the three mutant samples. Strong Spearman correlations shown suggest TE is generally unaffected by tRNA manipulation.Right: Spearman correlation, for each codon, between the ratio of mutant TE to wild-type TE and the percent of codon per gene. Significant correlations are shown as filled dots. For AGG mutants, the correlation is not higher for the manipulated codon (highlighted) than for other codons, indicating that optimizing codon usage does not affect TE. For ACA-K, the correlation is negative for the ACA codon, suggesting a mild effect.Source data are available online for this figure.

One of the major goals of codon optimization in biotechnology is an increase in protein yield. Studies done on transgenes expressed at a large fraction of cellular mRNA abundance report increased protein abundance when the mRNA was optimized for codon bias (Gustafsson *et al*, 2004; Lavner & Kotlar, 2005; Burgess-Brown *et al*, 2008), suggesting that codon usage contributes to efficiency (Supek & Smuc, 2010; Tuller *et al*, 2010b). However, other studies observed that optimizing codon adaptation of a reporter does not significantly improve TE or protein yield (Wu *et al*, 2004; Kudla *et al*, 2009; Welch *et al*, 2009; Hense *et al*, 2010; Letzring *et al*, 2010; Shah *et al*, 2013). Our experiments likewise provide support for the view that the TE of endogenous mRNAs is unchanged by effective codon optimization achieved by changes in the tRNA pool (Fig4). We find that increasing tRNA abundance or replacing the tRNA body sequence by one with higher tAI does not improve efficiency: Most genes remain unchanged in TE between the wild-type and mutant samples (Pearson *r* = 0.96 for AGG-OE and *r* = 0.95 for AGG-QC). Further, the top 200 genes that do deviate most in TE relative to the wild-type sample have mutant TE that is both lower (*reduced TE genes*) and higher (*increased TE genes*) compared to wild-type, with bias toward reduced TE genes (123 reduced versus 77 increased for AGG-OE and 133 versus 67 for AGG-QC). In AGG-OE, we observe no correlation between the fraction of AGG codons per message and the change between mutant and wild-type TE (Spearman *r* = 0.00002, *P* = 0.99); we would expect a positive correlation if increasing tRNA abundance increased TE. Further, despite the many-fold overexpression of tRNA, the correlation between TE and fraction of codon per message for AGG is not higher than the correlation for any of the other codons (Fig4). AGG-QC behaves similarly, such that manipulating the tRNA to be “faster” does not lead to a scenario where AGG outperforms other codons in affecting translation efficiency. Finally, these observations also hold if we look at protein synthesis rates instead of TE (Supplementary Fig S5).

While improving codon optimization by changes in tRNA structure or abundance does not seem to causally affect TE, we do see evidence for a modest impact from tRNA depletion (Fig4). Mutant and wild-type TEs are generally correlated in the ACA-K mutant (Pearson *r* = 0.96). Although there are more reduced TE genes than increased TE genes (127 versus 73), this difference is not significant via a permutation test (see Materials and Methods). However, we find a negative correlation, the lowest of all codons, between the fraction of ACA codons per message and the change in TE between mutant and wild-type (Spearman *r* = −0.08, *P* < 10^−8^), as we would expect if decreasing tRNA abundance decreases TE through a direct effect on its cognate codon. One explanation is that tRNA reduction could compromise TE if the demand is higher than the supply—the number of ACA occurrences in the genome is about the average number of occurrences over all codons, but we reduced its levels below those of any other tRNA. However, if protein synthesis and thus TE are controlled by initiation, this implies some feedback from slowed elongation on initiation, whereby affected ACA codons might stack ribosomes. In particular, reduced TE genes compared to increased TE genes have slower-than-expected codons closer to the 5′ end and stronger pausing in the first 100 codons (Supplementary Fig S6; significant under Kolmogorov–Smirnov test; see next section for definition of slower-than-expected codons as “outliers”). These confounding factors might contribute to the decrease in TE for ACA-heavy genes. Alternatively, ribosome stacking at ACA codons could induce fall off and reduced processivity that manifests as decreased TE.

To situate our results in the context of many previous studies on codon bias and tRNA abundance, we note that our observation focuses on endogenous messages with physiological or near-physiological tRNA levels. When the tRNA pool is limited compared to the number of free ribosomes, as in strong overexpression of transgenes, simulations indeed show that large demand for tRNAs can be rate-limiting (Chu *et al*, 2011; Chu & von der Haar, 2012; Shah *et al*, 2013). Experiments showing rate-limiting effects of tRNA abundance likely operated in this non-physiological regime. In addition, manipulation of codon usage rather than the tRNA abundance can perturb mRNA structure and other non-coding sequence features; our experiment is less susceptible to those issues.

### Factors for elongation efficiency

The notably modest effect of dramatic changes to the tRNA pool motivates the question: What signals do affect elongation efficiency and translation efficiency? We first take advantage of the ribosome profiling data to understand elongation efficiency—the time for a ribosome to finish translating a transcript once initiated—by studying rate-limiting elongation signals via inspection of outliers in the footprint counts. Based on the observed footprint counts and our model parameters for expected codon dwell time, we define *slow outliers* and *fast outliers* at each position *k* along a message *m* as positions where ribosomes are stalled more or less than expected, respectively. We denote their deviation from expected dwell time as *outlier strength* Δ_*mk*_ (see Materials and Methods). We considered a broad array of potential correlates of Δ_*mk*_, based on the literature hypothesizing their association with variation in codon translation rate or pausing, classified into eight categories (Supplementary Table S2): position on message, structure in downstream windows, protein folding, wobble base pairs, reuse of tRNAs from nearby codons, downstream RNA-binding protein motifs, nascent peptide effects, and global features. Supplementary Table S3 shows these correlations, which include significant features in the position, structure, wobble, and nascent peptide categories. We discuss these below and in Supplementary Note S1.

**Figure 5:**
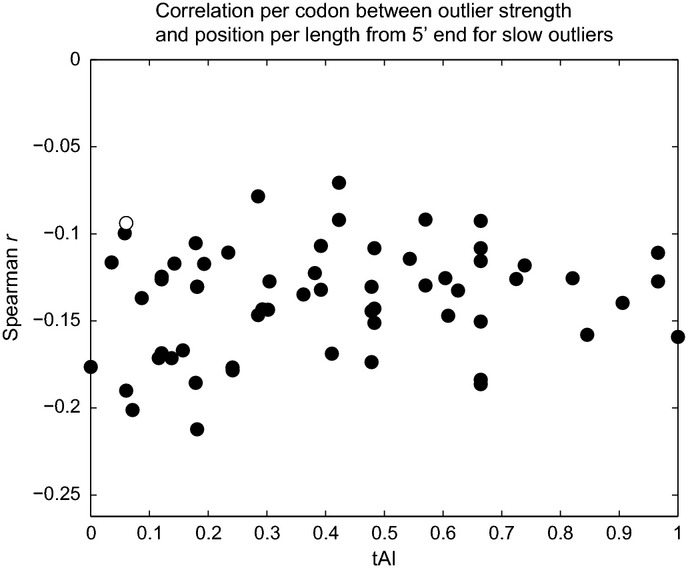
All codons show negative correlation between outlier strength and proximity to gene start Correlation between slow outlier strength and position per length from 5′ end, conditioned by the codon, plotted against codon tAI. For each codon *c*, we calculate the Spearman correlation for outlier strength Δ_*mk*_ and position per length from 5′ end (*k*/*L*_*m*_) but restricted to the (*m,k*) that satisfy *codon(m,k) = c*. All codons except one (hollow circle), which has the lowest abundance in the genome, have a significant negative correlation. This indicates that 5′ end outliers are slower even independent of codon bias.

The strongest correlation with outlier strength for slow outliers is proximity to the 5′ end, with larger pauses occurring closer to the beginning of a message, even relative to gene length or even when aligned by stop codon as opposed to start codon (position from 5′ correlates to Δ_*mk*_ with Spearman *r* = −0.043; position from 5′ per length with *r* = −0.144; and position from 3′ end with *r* = 0.162, *P* ≈ 0 for all). Similar observations of increased ribosome occupancy at the 5′ end have produced various hypotheses for the causal basis. In the “ramp” model (Tuller *et al*, 2010a), the presence of more slow codons (low tAI) at the beginning of a message is thought to separate ribosomes early to avoid the wasteful expenditure of resources on stacked, idling ribosomes. However, we observe a correlation between position from 5′ end and slow outlier strength even when conditioning on the codon (Fig5) and thereby controlling for differences in codon usage at different positions within the gene, suggesting that there is an initial low translation speed, regardless of codon usage, which gradually increases as translation proceeds. Additionally, our model helps account for length, position, and abundance biases when calculating outliers in a particular message in two ways: First, we include message-specific codon dwell times, and, second, we exclude the first 100 codons from each gene during model learning (see Materials and Methods) to avoid inflating or otherwise biasing the expected rates 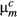 and μ^*c*^. Our analysis indicates that pausing occurs at the 5′ end, even after accounting for major factors such as codon bias and gene length.

Other explanatory signals have been suggested for pausing in ribosome profiling datasets (Stadler & Fire, 2011; Li *et al*, 2012; Charneski & Hurst, 2013). Our analysis shows a (mild) correlation between pausing and computationally predicted downstream mRNA secondary structure (Spearman *r* = 0.021, *P* ≈ 0 with structure measured by the density of stems). This correlation is reproduced when considering experimentally derived *in vivo* structure data from high-throughput DMS probing of unpaired A and C bases (Rouskin *et al*, 2013) (*r* = −0.033). It is also maintained when we restrict our analysis to slow outliers in the first 100 codons (*r* = 0.015 for density of stems and a similarly reduced *r* = −0.026 for *in vivo* energy, potentially due to genes with short UTRs and the decreased reliability of DMS structure probing data at ∼20 nt or less from the 5′ end), and so the effect is not necessarily caused by structure elsewhere on the strand. Single-molecule experiments with bacterial ribosomes (Chen *et al*, 2013) found that some hairpin and pseudo-knot constructs at varying distances downstream of the active codon can slow down the ribosome; structural energy could therefore potentially contribute to the excess ribosome density at the 5′ end. We also see a positive correlation on that same order of magnitude between slow outliers and the number of proline codons in the two sites upstream of the active codon (*r* = 0.069, *P* ≈ 0), as observed in other organisms (Ingolia *et al*, 2011; Woolstenhulme *et al*, 2013). Two correlations that we observed are not expected on the basis of previous studies. A study showing pausing specifically at CGA (Letzring *et al*, 2010) suggests slower elongation on wobble base pairs, whereas we observe the opposite correlation; this discrepancy might arise because the wobble effect is limited to a few specific codons, or to repeated wobble codons, or because of an incomplete characterization of codon/anticodon pairings which limits our assignment of wobble decoding. The correlation with charge observed by Charneski and Hurst (2013) holds in sign but not in significance even when considering the number of Arg and Lys residues in a window upstream of the active codon, although this result was later attributed to technical artifacts relating to the strand orientation (Charneski & Hurst, 2014).

### Factors correlating with translation efficiency

While elongation efficiency measures time required to synthesize a new protein, translation efficiency measures the throughput of protein synthesis. Besides codon adaptation, which we find to play little or no causal role in improving efficiency, other significant correlates to TE include structural features and the sequence motif around the start codon (Supplementary Fig S7).

**Figure 6:**
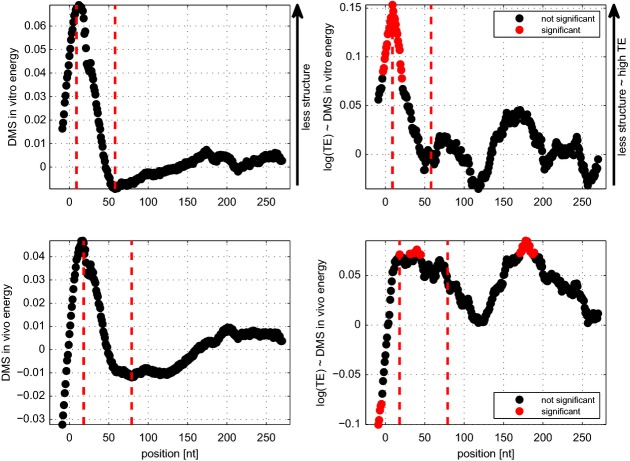
RNA structure energy and its relationship to translation efficiency Left: Energy averaged in sliding windows of 40 nt (see Materials and Methods) across all genes for *in vitro* and *in vivo* measures of energy via DMS probing (Rouskin *et al*, 2013). The second red line corresponds to the first window with lowest energy (˜60 nt for *in vitro* and ˜80 nt *in vivo*).Right: Spearman correlation between the energy windows and TE. The first red line corresponds to the first window with significant correlation (9 nt for *in vitro* and 18 nt for *in vivo*).

Structure is reduced near the translation start site in many organisms (Gu *et al*, 2010; Zhou & Wilke, 2011) and, in combination with specific structural motifs downstream, can promote or halt initiation (Kozak, 1990; Kochetov *et al*, 2007; Robbins-Pianka *et al*, 2010). We performed a sliding window analysis (see Materials and Methods and Fig6) to correlate TE with RNA secondary structure in 40 nt windows along the gene, for both experimental *in vitro* and *in vivo* structural energy (Rouskin *et al*, 2013). The window near the start codon is most significant, as reported previously for computational and *in vitro* structure measurements (Kudla *et al*, 2009; Kertesz *et al*, 2010; Tuller *et al*, 2010b; Keller *et al*, 2012); the positive correlation indicates that increased TE corresponds to loose structure in this region. Indeed, this is also the window with highest energy, corresponding to the lowest structure, as averaged over all genes (first red line in Fig6). Interestingly, the correlation with TE for *in vivo* structure is less pronounced and the window is shifted 3 codons downstream. We call this Window A.

Our attention was also drawn to the window downstream of the start codon at ∼60 nt *in vitro* and ∼80 nt *in vivo* (second red line in Fig6) with the lowest energy (more structure) compared to neighboring positions. We call this Window B. The most likely role for this energy barrier seems to be a stalling mechanism. Ribosome density is high nearby: At 132 nt (approximately two to three ribosome footprints downstream), our model-estimated ribosome density has a notable peak that is reduced when we exclude outliers, which capture positions where sufficient pausing could stack ribosomes (Supplementary Fig S8). Although properly placed downstream structure can improve the efficiency of initiation by stalling the scanning pre-initiation complex (Robbins-Pianka *et al*, 2010) or might be selected for heavy structure in order to prevent other regions (namely around the start codon) from being paired, the lack of significant correlation with TE for Window B suggests that ribosome flow control here optimizes other aspects of translation besides throughput.

**Figure 7:**
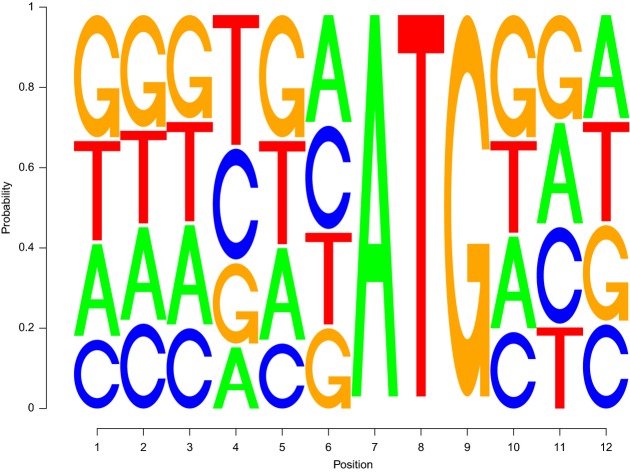
Estimated Kozak motif for efficient genes Estimated TE-driven Kozak motif based on a regression model (see Materials and Methods). The original Kozak consensus for yeast (Hamilton *et al*, 1987) is WAMAMAATGTCY.

In addition to low structure at the start codon, initiation may be assisted by recognition of a 12-mer motif around the start codon called the Kozak sequence in eukaryotes (Kozak, 1981), derived in yeast based on a sequence consensus from highly expressed genes by Hamilton *et al* (1987). As expected, due to a tight correlation between mRNA abundance and TE (Supplementary Fig S7), similarity to the Kozak motif correlates strongly to TE (Spearman *r* = −0.21, *P* < 10^−45^) (measuring similarity by Kullback–Leibler divergence to the position weight matrix where 0 divergence means a closer match). The 3^rd^ nucleotide preceding AUG is the most significant (Spearman *r* = −0.17, *P* < 10^−29^), consistent with experimental measures of initiation efficiency after modifying positions in the Kozak site (Looman & Kuivenhoven, 1993; Yun *et al*, 1996). Using a linear regression model for predicting TE based on a set of correlates suggested in the literature (see Materials and Methods), we learn a refined Kozak motif to reflect highly *efficient* genes (Fig7). Our learned Kozak motif reduces the error of our regression model predictions relative to an equivalent model using the original motif (from 0.84 to 0.75, averaged over 100 test sets selected randomly, compared to a null model error of 0.97) (Supplementary Table S4). This indicates that our refined motif better corresponds to highly translated genes, likely because it was trained directly on translation efficiency measurements rather than on a proxy such as mRNA abundance.

Finally, we tested the correlation between translation efficiency and other mRNA features often discussed in the literature (Supplementary Fig S7). We find a negative correlation with evolutionary rate that is suggestive of the intuitive fact that more conserved genes are more highly translated. The positive correlation we find with mRNA abundance suggests a model of co-expression where the need for high protein abundance drives high translation of abundant transcripts. Consistent with previous studies (Ingolia *et al*, 2009), we observe a very small negative correlation with length. We also find a positive correlation (although weaker than that for tAI) to the codon translation rates geometrically averaged over the codons within a gene. Lastly, RNA-binding proteins (RBPs) have recently received attention for their roles in post-transcription regulation, and we also see high Spearman correlations between RBP occupancy and TE. When looking at enrichment of 15 proteins, we find the expected correlation with translation efficiency (as suggested by the literature) in eight of ten cases. One of the two “unexpected” proteins, scp160, was recently reported to be required for translational efficiency of particular mRNAs in yeast (Hirschmann *et al*, 2014), even though it correlates negatively to ribosome occupancy in Hogan *et al* (2008); our analysis encouragingly suggests the former correlation. Supplementary Note S1 has further discussion.

## Discussion

In this paper, we present a statistical model to extract codon translation rates and protein synthesis levels from ribosome profiling data. This robust framework allows us to shed new light on causality in regulation of translation and characterize the features associated with efficient elongation and translation. Although codon usage is a strong correlate to TE (Supplementary Fig S7), our mutant experiments suggest (via the correlation between codon bias and tRNA abundance) that codon usage may not causally influence efficiency. The direct impact of codon usage on efficiency and the basis of the selective force underlying codon bias has remained a topic of controversy for decades. Some authors have proposed that codon optimization serves directly to enhance the translational efficiency of specific genes, perhaps by speeding elongation on their mRNAs. Our work provides direct experimental evidence against this view. Rather, our work is consistent with an alternative model, aligned with previous results for *Escherichia coli* (Kudla *et al*, 2009), in which codon bias in highly translated genes results from selection to optimize utilization of the translational machinery, whose abundance and production represents a major limitation on cell growth (Andersson & Kurland, 1990; Bulmer, 1991; Kudla *et al*, 2009); this selection induces a correlation without implying that increasing codon bias optimizes efficiency on individual genes (Welch *et al*, 2009). In this view, initiation is rate-limiting and thereby determines translational efficiency. When the demand–supply balance for a tRNA is not compromised by extremely high expression of a transgene not adapted to the host organism, we propose that selective forces beyond the TEs of individual messages guide the distribution of codons. The positive correlation between elongation rate and TE suggests a potential contributor, namely, selection for efficient use of ribosomes and translation factors, and that this selective force is strongest for high-expression, high-TE genes. Such selection pressure is consistent with studies of overall cell growth and protein synthesis, which indicate that the translational apparatus is rate-limiting for cell growth and that reduction in the amount of ribosome time devoted to producing an abundant protein can speed cell growth (Andersson & Kurland, 1990; Arava *et al*, 2003; Kudla *et al*, 2009; Mitarai & Pedersen, 2013). As elongation rate is not the strongest correlate to TE, other mechanisms also deserve further study. For example, there may be selective pressures on the mRNA sequence itself (e.g., to induce certain secondary structures), which in turn create pressure in the cell to ensure a sufficient supply of tRNAs for efficient translation of the highly translated messages. Our results are also consistent with the prevalent view that initiation is typically the rate-limiting step in protein synthesis, which does not provide a clear mechanism for codon usage in the body of a gene to affect its efficiency, and particularly not through increased elongation rates. Instead, tRNA levels are likely forced to match the lack of disfavored codons by selection against the cost of tRNA production or against poor decoding accuracy.

Our model is designed to account for the complexities of ribosome profiling data while keeping parameter estimation tractable. Although average footprint density on a gene is well correlated to protein abundance, outliers can pull the estimate provided by the mean away from the true level, especially when ribosome stacking is common. Thus, properly accounting for differential elongation rates can improve inference of protein synthesis levels from this data. We maintain a simple translation model (for example, we do not explicitly include a rate of ribosome falloff or an analytical treatment of codons being processed in series), but our design choices trade-off for model simplicity, algorithmic stability, and smoothing of noisy data. Using one model parameter for all codon instances in a gene, as opposed to an individual dwell per position, has several advantages: It averages out sequence biases in footprint fragments, makes the optimization algorithm less susceptible to local minima and hence robust to parameter initialization, and allows us to infer parameters even for low abundance genes by offsetting the lack of data with soft prior constraints. We reassuringly find qualitatively similar results when we replace our refined protein synthesis rates with a simple average of the footprints per gene, while obtaining better quantitative estimates compared to existing protein abundance datasets. More physics-based or simulation models (Zhang *et al*, 1994; Reuveni *et al*, 2011; Tuller *et al*, 2011) require knowledge of the kinetic parameters of translation, can necessitate grossly simplifying assumptions such as a single codon translation rate per gene, base certain model quantities on a limited set of features, or directly assume that codon rate is correlated to codon adaptation. In comparison, our method reduces the number of assumptions made by directly modeling the experimental processing and fitting the model parameters to the data under the single concept of flow conservation. On the other hand, methods that aggregate the data directly (Qian *et al*, 2012; Charneski & Hurst, 2013; Gardin *et al*, 2014), similar to our baseline method for calculating codon translation rates, do not readily lend themselves to computing other quantities. For example, because we have an underlying model, detection of outlier codon positions follows easily within our framework, whereas other works rely on choosing an adjacent window of appropriate size to compare counts. Similarly, we can easily study other potentially interesting effects, such as codon translation rate variance within genes and among genes. Finally, our method would particularly be useful in situations where ribosomal profiling data are scarce or noisy. By using a probabilistic model, we infer rates of interest from the observed, noisy data without needing to exclude genes with sparse information. With the growing usage of ribosome profiling, a robust framework for studying rates of elongation and synthesis is essential.

Our resulting analyses address the contributions of initiation versus elongation to efficiency (Arava *et al*, 2003; Lackner & Bahler, 2008; Shah *et al*, 2013). While efficient usage of ribosomes and elongation factors influence the overall amount of protein produced from the whole genome, initiation may dictate differences between genes (Firczuk *et al*, 2013). We characterize two initiation signals that could play a role in translation regulation via a two-stage metering-light model: reduced structure around the start codon and favorable sequence context to promote ribosome binding, followed by an increase in structure that could, in turn, serve to reduce misfolding of the emergent polypeptide by allowing sufficient time for recruitment of chaperones to the ribosome exit tunnel (Fredrick & Ibba, 2010). This barrier could reflect the observed universal per-gene effect, independent of codon identity, whereby the strengths of slow outlier positions correlate to 5′ end proximity. Since translation is resource heavy, requiring tRNAs, mRNAs, and ribosomes, with the latter being especially costly to produce, we intuit that the cell must balance the use of these finite resources while at the same time producing functional protein products. Structure around the 5′ end could be one of the key mechanisms through which the cell regulates translation so as to avoid wasting resources.

The region of slow elongation at the 5′ end certainly merits further exploration. In contrast to the slow-codon ramp proposed in Tuller *et al* (2010a), our model shows that while there may be an abundance of low-tAI codons near the 5′ end, these codons do not cause slow elongation (Supplementary Fig S9). We find (mild) correlations between pausing and downstream structure, between tAI and downstream structure over the first 50 codons of all genes (Spearman *r* = −0.0055, *P* = 0.01 for stem density, and insignificant for *in vitro* or *in vivo* structure), but not between codon usage and codon translation rate. A study performed over diverse bacteria, controlling for GC content, proposes that structure drives codon usage early at the 5′ end (Bentele *et al*, 2013); in yeast, there may be similar selection whereby structure-related constraints induce a low-tAI ramp.

The impact of secondary structure on translation is complex. In addition to a role in initiation, high structure regions could also act by influencing elongation (Chen *et al*, 2013). Outliers in the high-variance ribosome profiling data can differ from expected dwell times by a factor of 40 and are distributed throughout the message (Supplementary Fig S10). One explanation is the presence of downstream structural features that create an energy barrier to elongation; these correlate (more weakly) to outlier strength when ignoring the first 100 codons (whole gene versus truncated gene has *r* = −0.033 versus *r* = −0.034 for downstream *in vivo* energy and *r* = 0.021 versus *r* = 0.010 for density of stems), precluding the possibility that high ribosome density (based on the 5′ end as a proxy) drives the effect. In addition, mRNA-binding factors can interact with structure (Dethoff *et al*, 2012), but whether structure performs any common genome-wide functions is not yet established. One possibility is that secondary structure slows the ribosome during elongation to promote correct folding of the nascent protein during its vectorial synthesis by the ribosome.

The significant but mild correlation with structure suggests that other factors also play important roles in pausing. Experiments suggest that the wobble base in the CGA codon causes significant pausing (Letzring *et al*, 2010; Stadler & Fire, 2011), clusters of slowly translated codons could stall ribosomes more than the sum of their individual decoding times (Zhang *et al*, 2009), and effects from the nascent peptide could stall elongation, for instance at prolines (Ingolia *et al*, 2011; Woolstenhulme *et al*, 2013). It is likely that a compendium of biological features interact to dictate elongation rate. Although our genome-wide outlier analysis shows promising correlations between pausing and features, the small magnitude of the correlation could be improved by looking at more restrictive or genetically meaningful sets of positions. The growing interest in ribosome profiling poses exciting directions for further investigation of the interactions between these features and the changes that may occur in different conditions. With this additional data and measurements from single-molecule experiments (Wen *et al*, 2008; Uemura *et al*, 2010), our model could be extended to include finer-grained parameters for codon translation rates, partitioned in various ways, in order to better understand how rate changes over a transcript. Further analysis is also needed into how structure and the sequence around the initiation site work together or against each other. For example, heavy structure can promote initiation in spite of weak initiation context, but the ways that they interact are still unknown.

In this paper, we present a method that provides a rigorous perspective for analyzing the increasing number of ribosome profiling datasets and thereby addressing these important questions. We illustrate the use of the method in the context of one of these datasets to create a high-level view of the mechanisms involved in initiation and elongation, to study the factors affecting initiation as the rate-limiting step for translation, and to support a model in which the direction of causality goes from translation efficiency to codon usage rather than the opposite.

## Materials and Methods

### Ribosome profiling datasets

All experiments were done on yeast strain 288C. Cells were collected for ribosome profiling by filtering ∼250 ml culture of OD = 0.6 and immediately flash freezing on liquid nitrogen. For all ribosome profiling experiments, footprints were obtained as described before (Ingolia *et al*, 2012). Three out of four copies of threonine tRNA (tT(UGU)G2, tT(UGU)H, tT(UGU)P), recognizing the ACA codon, were knocked out using the standard technique of homologous recombination from a plasmid PCR product. The resulting strain was marked with nourseothricin, kanamycin, and hygromycin B resistance, respectively. Successfully transformed yeast were identified by check PCR. tRNA arginine (tR(CCU)J) recognizing the AGG codon was overexpressed by cloning into a URA marked 2-micron plasmid (pRS426) and transforming wild-type yeast using –URA selection. For the tRNA body swap, tRNA sequence from tR(UCU)B was mutated in the anticodon to CCU using QuikChange Site-Directed Mutagenesis kit (Stratagene) in order for the tRNA product from tR(UCU)B to recognize the AGG codon. The mutated tRNA was then cloned in the 2-micron plasmid pRS426 and transformed into 288C.

Ribosome-protected fragments were aligned against *Saccharomyces cerevisiae* assembly R63 from the Saccharomyces Genome Database (SGD, http://www.yeastgenome.org), and we kept uniquely mapped reads with no more than two mismatches and lengths between 28 and 31. To identify the active codon for ribosome-protected fragments, we let 0 be the first nucleotide of the read and if the read begins on the first/last/middle nucleotide of a codon, the active codon starts at nucleotide 15/16/17, respectively. An mRNA fragment was mapped to a gene if it begins < 16 nt upstream of the start codon and more than 16 nt upstream of the stop codon. Genes were ignored if they did not have an AUG start codon, had internal stop codons, had < 50% of positions on the coding sequence with at least one mapped mRNA count, or if all the footprint counts were 0 over the gene length used in the translation model (see below), leaving around 5,000 genes in each sample. When comparing mutants to wild-type samples, we used the intersection of the valid genes in each sample. The AGG mutants were compared against the wild-type sample with a URA plasmid.

### Analysis of tRNA charging and relative RNA levels

For analysis of charging levels of tRNAs, duplicate samples of each strain were grown under conditions used for ribosome profiling, followed by harvesting of ∼4 OD-ml of cells. Then, bulk RNA was prepared from each pellet under acidic conditions (pH 4.5) using glass beads, and RNA was resolved on a 6.5% acrylamide gel at pH 5 for 15 h at 4°C, transferred to Hybond N+ membrane, and hybridized with appropriate 5′-labeled oligonucleotide probes, as described (Alexandrov *et al*, 2006). Charging levels were visualized on a Typhoon PhosphorImager (GE Healthcare) and quantified using ImageQuant, and relative levels of tRNA^Arg(CCU)^ were measured by normalization to levels of tRNA^Leu(CAA)^ in the corresponding lane.

### Feature calculations

Gene copy numbers for tRNA were obtained from the tRNAscan-SE database (Lowe & Eddy, 1997). To measure codon usage bias, we use tAI, which ranges from 0 to 1 for more preferred codons, calculated as in Dos Reis *et al* (2004) with refined weights described in Tuller *et al* (2010a).

Experimentally derived structure data from DMS probing (Rouskin *et al*, 2013) were normalized in windows of size 150 nt by the minimum count in the top 5% of A and C nucleotides, and the top 5% of counts were set to 1. Windows with < 10 A and C nucleotides in the top 5%, windows with a zero normalization constant, genes without data, and genes without a characterized UTR (Nagalakshmi *et al*, 2008) were ignored in analyses. In the sliding window energy analysis, energy windows were normalized per gene by the mean over windows on each gene. In the energy profile, normalized windows were then averaged across positions without missing data, aligned by start codon. In the energy–TE correlation profile, we applied a conservative Bonferroni correction by multiplying the *P*-values by the number of windows (30 upstream of the start codon and 250 downstream, since this span covered the maximum number of genes). To calculate the location of the dip in the energy profile, we identified global minimums within spans of 90 nt and took the first minimum.

The correlation between tAI and downstream energy is for tAI over windows of three codons in the first 50 codons of all genes and the associated average of the 40 nt energy windows 15 nt downstream from each nucleotide in the tAI window. Energy windows are calculated as above using the number of stems and DMS *in vitro* and *in vivo* energy.

### Translation model

As discussed in the main text, we optimize our objective over the parameters 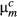 and μ^*c*^ and solve for *J*_*m*_. Since individual footprint counts can be noisy and sparse, we smooth the data in three ways. First, we use a single 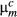 for every copy of codon *c* on message *m*. The dwells 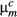 for a specific *c* over all genes *m* softly agree with the global μ^*c*^ in a weighted geometric average with weight w_*m*_^*c*^: the number of codons *c* on gene *m* normalized by the number of codons *c* over all genes. Hence, genes with more copies of codon *c* get a larger vote in the average estimating μ^*c*^. Second, we add a pseudo-count of 1 to all footprint counts and use the logarithm of normalized counts in the Poisson term (similar to a more robust geometric average as opposed to an arithmetic average that is easily skewed by outliers), first scaling the flow-normalized counts by a single factor over all (*m,k*) so that the lowest one is 1. We refer to these transformed counts as *d*'. Third, during model training, we ignore the first 100 codons (or the first 25% for genes shorter than 100 codons) since this region may have unusual flow conservation properties. If it does not, excluding these codons should not affect the learned rates. We refer to these restricted positions as *k*'. The second term in the objective function is multiplied by a constant *C* = 100 so as to not be greatly outweighed by the data term. Altogether, we solve the following optimization problem (where *k'* is restricted and *d'* are scaled as described above): 

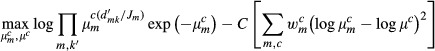


We verified that the constant *C* did not affect our results by running the main analyses again—correlations for codon bias measures, protein abundance, and outliers—on several other values (1, 10, 1,000, 10,000, 100,000). We note no significant change (Supplementary Table S5), except for some outlier correlations for 100,000 (stemsGC-down15 is now not significant; cluster-ArgLys-up-1 is significant) and for 1 and 10 (internal-down is now significant). Similar to taking the limit of the constant to infinity, we also considered a model with only μ^*c*^ parameters and no 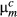 (and hence no second term in the objective function) (Supplementary Table S5). Again, no extreme change exists in the correlation between codon translation rate and codon bias measures. Perhaps because we have removed a layer of parameters, we do see a slight decrease in correlation with protein abundance and some changes to outlier correlations: Multi-down is no longer significant but still shows a similar correlation strength; is-in-domain is significant, suggesting that slow outliers lie outside of protein domains, and the upstream number of Arg/Lys codons is now significant.

The optimization algorithm is as follows: *J*_*m*_ is fixed to *D*_*m*_ = Σ_*k in m*_ *d*_*mk*_/*L*_*m*_ and 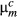 and μ^*c*^ are initialized to dwells from the baseline method (see below), shifted in log space so that the mean is log(7.2), plus a small random number. The value 7.2 is the mean over all (*m,k*) of the flow-normalized counts normalized and smoothed as described above for the wild-type sample. The appropriate mean value was replaced for each of the mutant samples. The parameters are estimated via coordinate descent by iterating through codons *c* and learning the associated 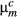 and μ^*c*^. Optimization per *c* used an L-BFGS method (Byrd *et al*, 1995; Matlab wrapper, http://www.cs.toronto.edu/∼liam/software.shtml) with the following stopping criteria: max number of iterations 5,000; gradient tolerance 1e-5; and function tolerance 1e3. Coordinate descent was stopped when the difference in weights was < 5e-5 or the difference in function value was < 1e-5. Codons not appearing in a particular gene *m* did not have an associated 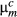, and we also excluded the stop codons. We then compute *J*_*m*_ = Σ_*k in m*_ (*d*_*mk*_/μ_*mk*_)/*L*_*m*_ = Σ_*k in m*_ (*d*_*mk*_/μ_*m*_^*c = codon(m.k)*^)/*L*_*m*_. The optimization is not sensitive to initialization (Supplementary Fig S11).

Although less robust, we also optimized a model with a separate dwell time μ_*mk*_ for every (*m,k*) with the following initialization of weights: μ_*mk*_ = d_*mk*_/*D*_*m*_, with 0 counts replaced by the mean of all non-zero counts, shifted in log space so that the mean is log(7.2); μ^*c*^ are dwells from the baseline method (see below) shifted in log space so that the mean is log(7.2); all weights perturbed by a small random value. The value 7.2 was chosen as above. L-BFGS settings were as above. Coordinate descent was stopped when the difference in weights was < 1e-2 or the difference in function value was < 1e-1. The overall codon dwell times μ^*c*^ were well correlated to those in the original model (Pearson *r* = 0.99, *P* < 10^−74^), but analyses based on dwell times per (*m,k*) could be impacted, since these parameters are more sensitive to initialization. So we verified all qualitative observations presented still hold. The correlation between codon translation rate and codon bias measures is insignificant (*r* = 0.151, *P* = 0.359 for Cy5; *r* = 0.138, *P* = 0.401 for Cy3; *r* = 0.223, *P* = 0.084 for tAI). Protein abundance estimates correlate similarly to external measures (*r* = 0.671 for De Godoy *et al* (2008) data and *r* = 0.778 for Newman *et al* (2006) data, *P* = 0 for both). In the outlier analysis, all correlations still hold except for the structure features only the density of stems 12 nt and 9 nt downstream are significant but the others are on the same order of magnitude, the protein domain feature is significant for bases inside a domain, and the feature for upstream number of Arg/Lys codons is significant. Correlations between TE and gene-level features are similar except Kozak position 2 is now barely not significant, experimental *in vitro* energy for the mRNA sequence is barely not significant, and Npl3 is significant (in the expected direction). The energy–TE correlation profile is the same except the window at 18 nt for *in vivo* energy is barely not significant but still a peak. The ribosome density graph has the same peak at 132 nt and decreases when outliers are removed. The refined Kozak motif has the same dominant bases except position 1 in Fig7 has the non-dominant *T* swapped with A. Finally, the error when replacing the learned Kozak motif with the original similarly increases from 0.69 to 0.77.

### Baseline method for codon translation rate

To get dwell time per codon *c* from the raw data, we average over counts *(m,k)* for which *codon(m,k) = c*, normalized by the average per gene (*D*_*m*_ = Σ_*k in m*_ *d*_*mk*_/*L*_*m*_). Rate is the reciprocal of dwell time. As above, we first add a pseudo-count of one to each *d*_*mk*_ and ignore the first 100 codons (or the first 25% for genes shorter than 100).

### Analysis of translation efficiency in mutants

To test whether the difference in the number of reduced TE genes versus increased TE genes (127 versus 73) in ACA-K is significant, we permuted the mutant TE values 1,000 times and calculated the number of reduced TE versus increased TE genes for each permutation. There were 0 cases where the difference was less than the original difference, indicating the original difference is not statistically significant.

### Model for translation efficiency

We used a regression model to predict TE of an mRNA message based on various features: 

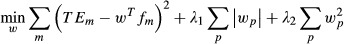


The first term fits an optimal set of weights *w* to the TE of a set of genes *{m}* using a linear combination of the set of features *f*_*m*_. The last two terms enforce sparsity (so that features that do not explain the data well receive a weight of 0) and shrinkage (so that weights are kept at a small scale). Under a standard machine learning framework, we divide the genes in our yeast dataset into a test set (size 400 genes) and a training set (the remaining genes). The hyperparameters λ_1_ and λ_2_ are learned via cross-validation: We further divide the training set into fifths and evaluate the error for a grid of hyperparameter values on each fifth of the training set. The weights *w* are then learned on the whole training set with the best hyperparameters (with lowest cross-validation error). Test set error is the squared norm difference between predicted and actual TE, averaged over all genes in the test set. For reference, we create a null model where the weights are learned from TEs randomly permuted among the genes. The final weights are the average over all training/test combinations. The features used are minimal in order to maximize the number of genes that have these characterized: tAI of gene; computationally predicted energy of 5′ UTR, 3′ UTR, mRNA, and window around the start codon with highest correlation with TE; length of coding sequence; mRNA abundance; and identity of bases overlapping the Kozac site [genes without a characterized UTR (Nagalakshmi *et al*, 2008) were excluded].

To compute the weights for the refined Kozak site, we include a feature *f*^*k*^ in *f* defined as *f*^*k*^ = 1/(1 + exp(x*g)). The vector *g* has 36 indicators, four per each of the nine positions in the Kozak site (excludes the start codon). The vector *x* has the corresponding weights for each indicator, is included in the shrinkage term, and is learned iteratively with *w*. The refined Kozak motif in Fig7 is the average of the 100 values of *x* learned separately for each training set. To create a position weight matrix from these weights, we shift the weights for each position so that the most negative value (if any) is 0 and normalize by the sum of the four weights at that position. The sequence logo was generated by seqLogo (Bembom O, seqLogo: Sequence logos for DNA sequence alignments, R package v1.28.0, http://bioconductor.org/packages/release/bioc/html/seqLogo.html).

To test whether the refined motif provides better TE predictions than the original Kozak motif, within each of 100 training sets, we fix *f*^*k*^ for each sequence with *x* set to the original motif (scaling the weights so that the sum at each position matches the sum of the learned motif) and learn the remaining weights as before. We then compute accuracy on the corresponding test set.

### Outlier model

The strength of an outlier Δ_*mk*_ at position *(m,k)* is defined as the difference between the observed count (*d*_*mk*_) and the expected count (*J*_*mk*_ * 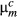), divided by *s*_*mk*_, a standard deviation representing the variance in that count due to the abundance of the gene and the codon it corresponds to. For *s*_*mk*_, we divide the genes into 32 quantiles by abundance and compute the standard deviation of the counts in each bin per codon. Thirty-two was chosen as the maximum number that still gave at least three counts in each bin per codon and no zero-valued *s*_*mk*_. This normalization helps distinguish true biological outliers from outliers arising due to differential mRNA sampling and abundance depths across genes. Counts are as in the optimization setup (*d*_*mk*_ have a pseudo-count of 1 and *J*_*mk*_ are scaled by a single factor). A slow outlier is an *(m,k)* with Δ_*mk*_* *> *T* for some threshold *T*. Non-outliers are *(m,k)* with −1 < Δ_*mk*_* *< 1, excluding slow outliers.

Since there is a small uncertainty in the position of the active codon within ribosome-protected fragments of certain lengths, what we might see as a fast outlier (a position *(m,k)* where Δ_*mk*_* *< −*T* and, for example, a wrongly labeled count of 0) could actually have a fragment that was falsely associated with an adjacent slow position. The opposite is much less likely; an observed slow outlier has many more counts than expected, making it unlikely that so many fragments were wrongly attributed and belong instead to an adjacent fast outlier. For that reason, we compare slow outliers only to non-outliers.

When correlating features to outlier strength (Supplementary Table S3), we call features significant only if they pass a stringent set of conditions: Pearson and Spearman correlations must have the same sign for all slow outlier thresholds (*T* = 0, 0.5, 1, 1.5, 2, 2.5) and be significant; the correlation when binned by codons must have at least 30 significant codons; the sign of the correlation must match the direction suggested by the comparison of means for slow versus non-outliers. When referring to significant features in Supplementary Table S3, we cite the correlation for *T* = 0 since all thresholds are significant. For a more stringent set of outliers, we use *T* = 1 in analyses requiring a fixed *T* (Supplementary Figs S6, S8 and S10).

### Accession numbers

Data are available through GEO Series accession number GSE63789.

## Supporting Information

Supplementary Table S5

Supplementary Note S1

Source Data for Supplementary Figure S2

Review Process File
