# Supplementary figures and images for: Causal signals between codon bias, mRNA structure, and the efficiency of translation and elongation
Source: Mol Syst Biol. 2014 Dec 23;10(12):770. doi: 10.15252/msb.20145524 (PMC4300493; doi:10.15252/msb.20145524)

Pearson:  $r=0.7885$

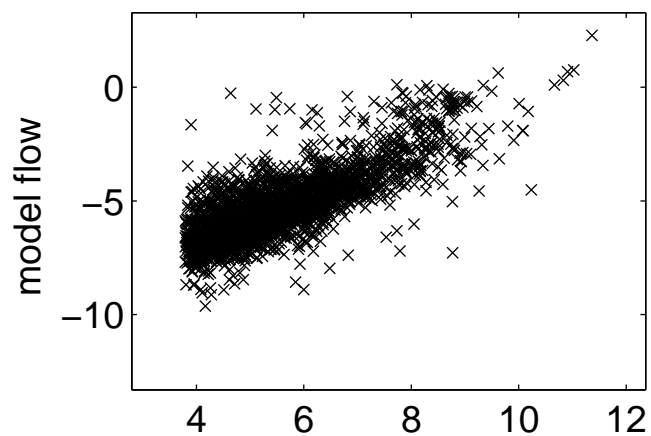

Pearson:  $r=0.6802$

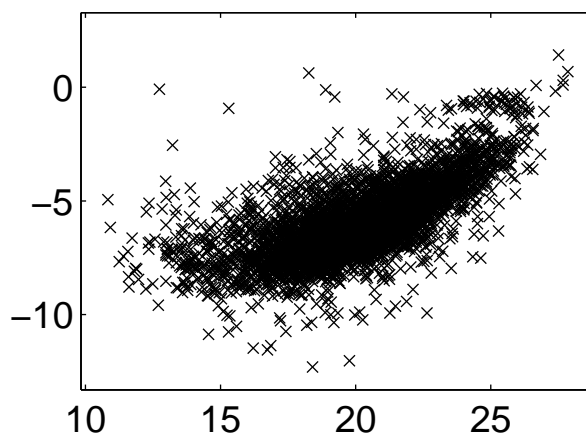

Pearson:  $r=0.7755$

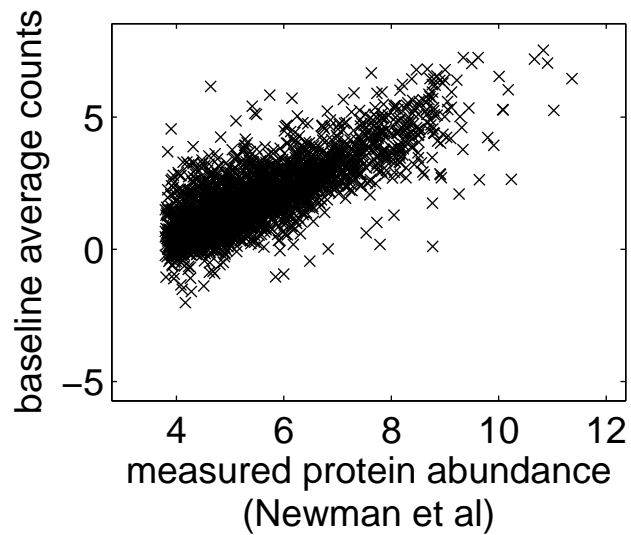

Pearson:  $r=0.6704$

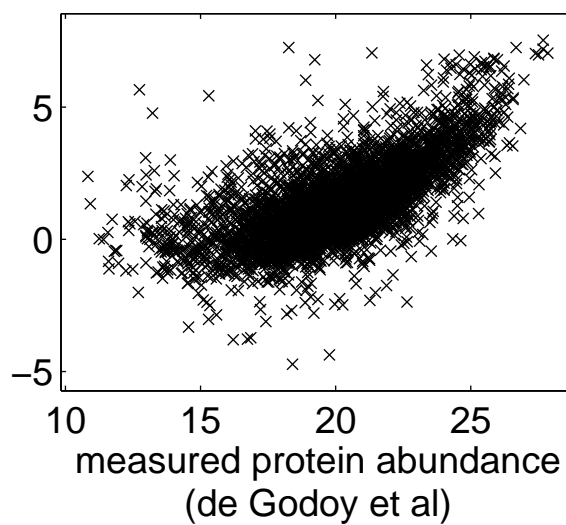

Supplement: Supplementary file 1 [file msb0010-0770-sd1.pdf]

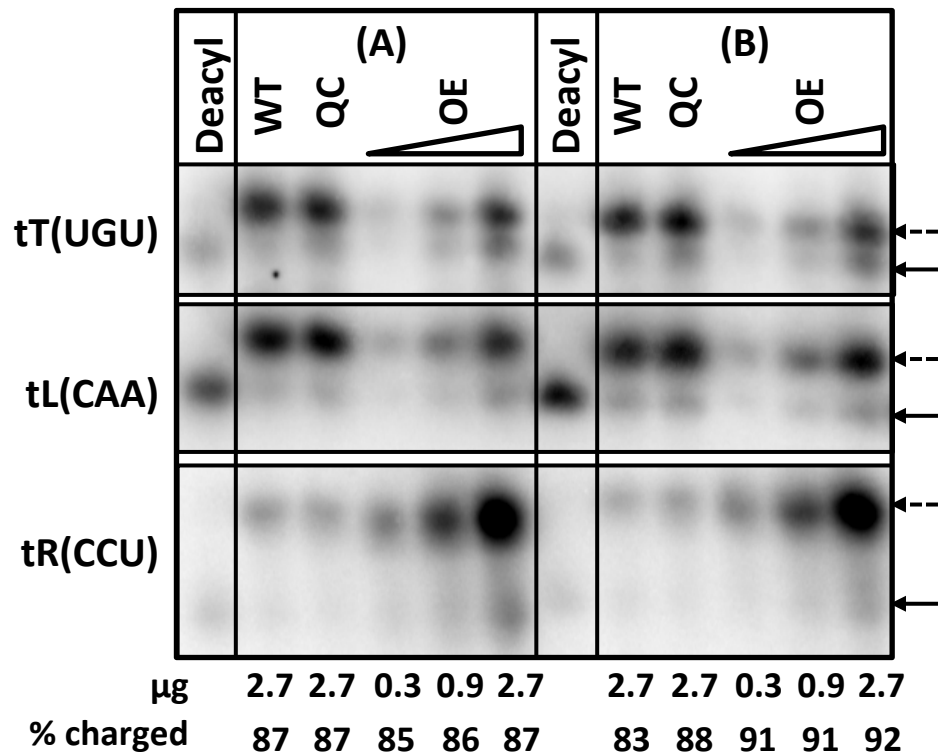

Supplement: Supplementary file 2 [file msb0010-0770-sd2.pdf]

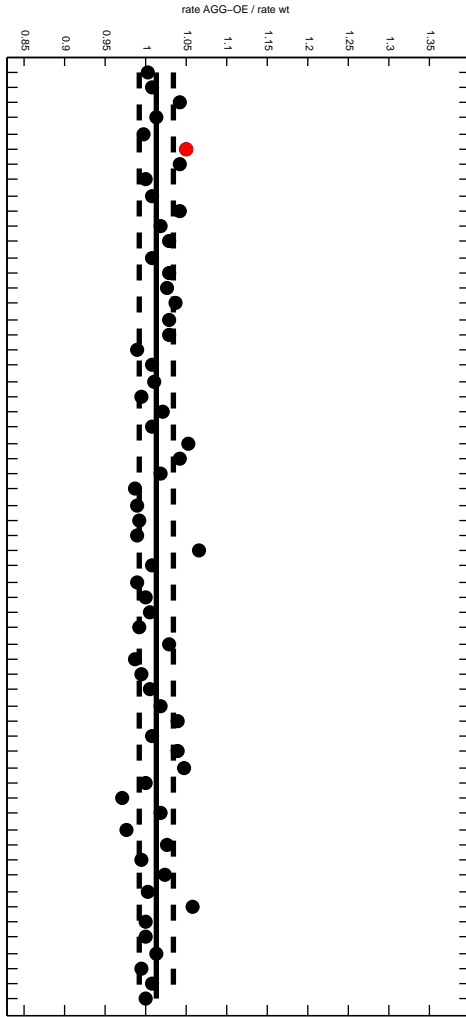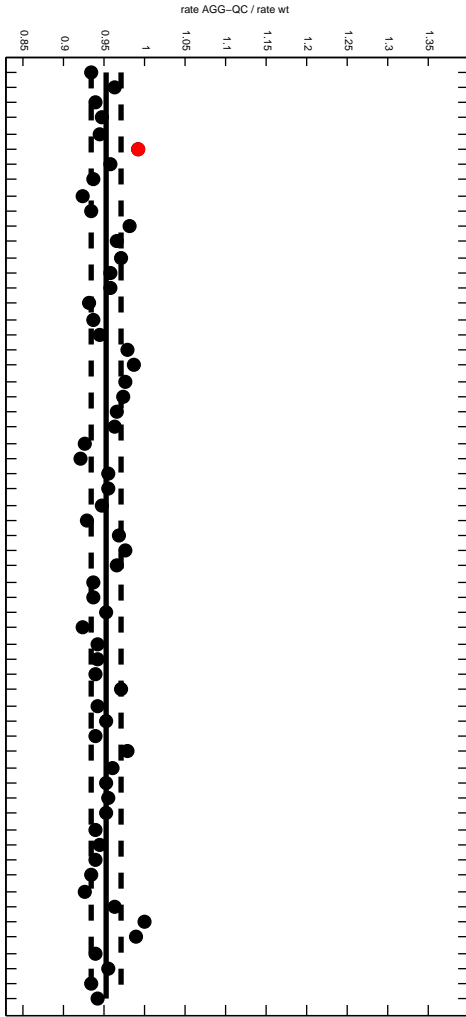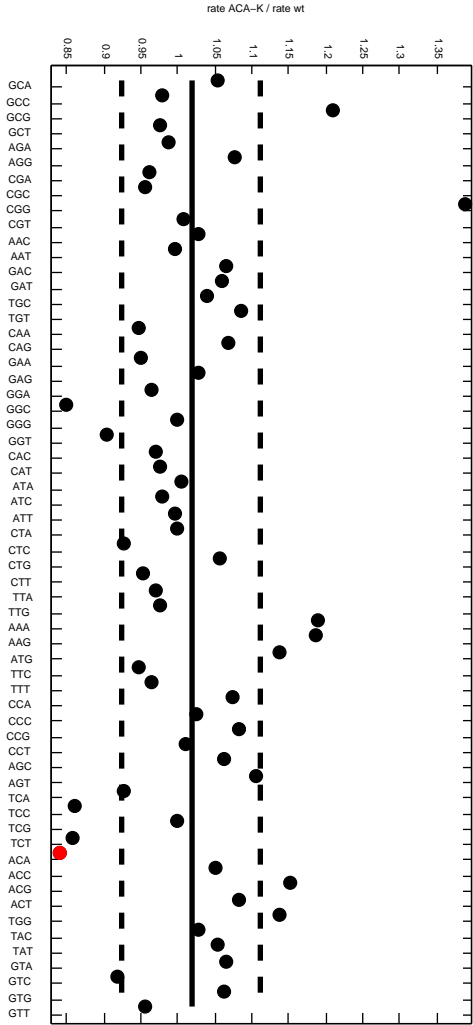

Supplement: Supplementary file 3 [file msb0010-0770-sd3.pdf]

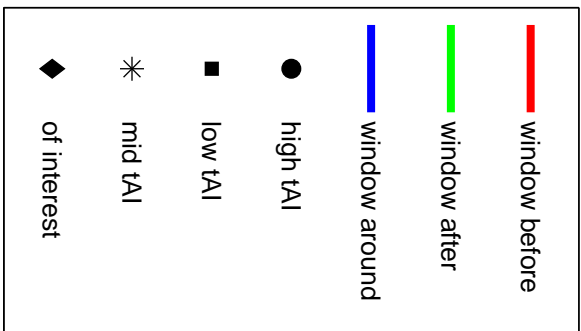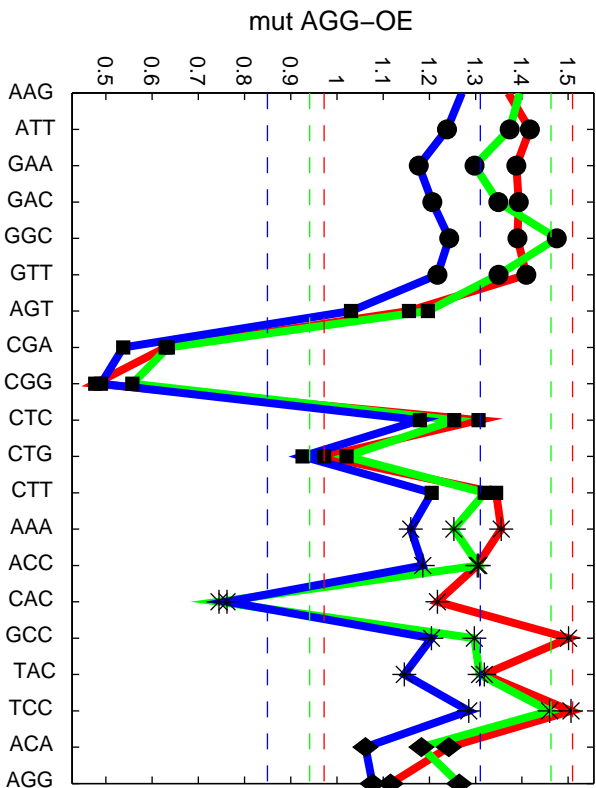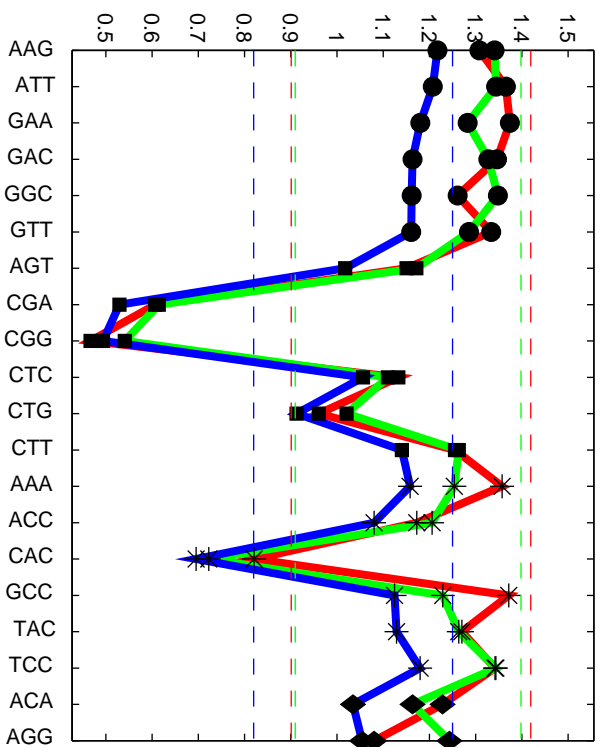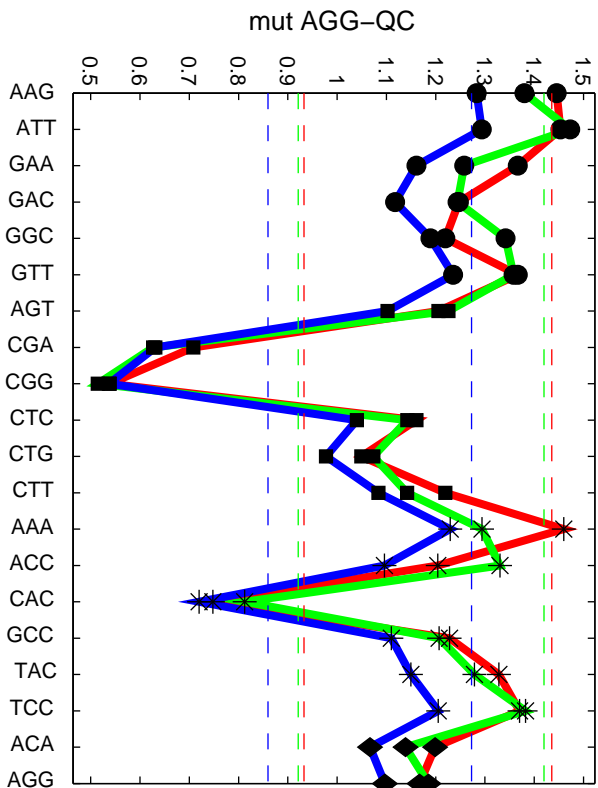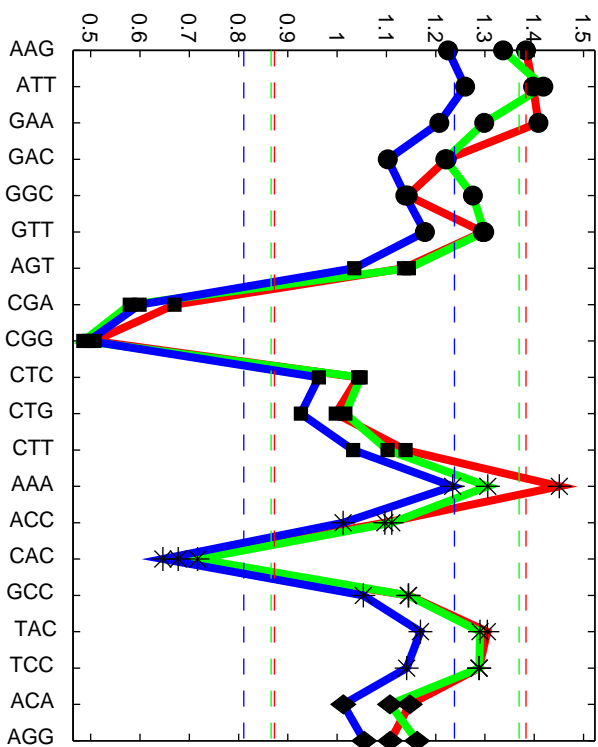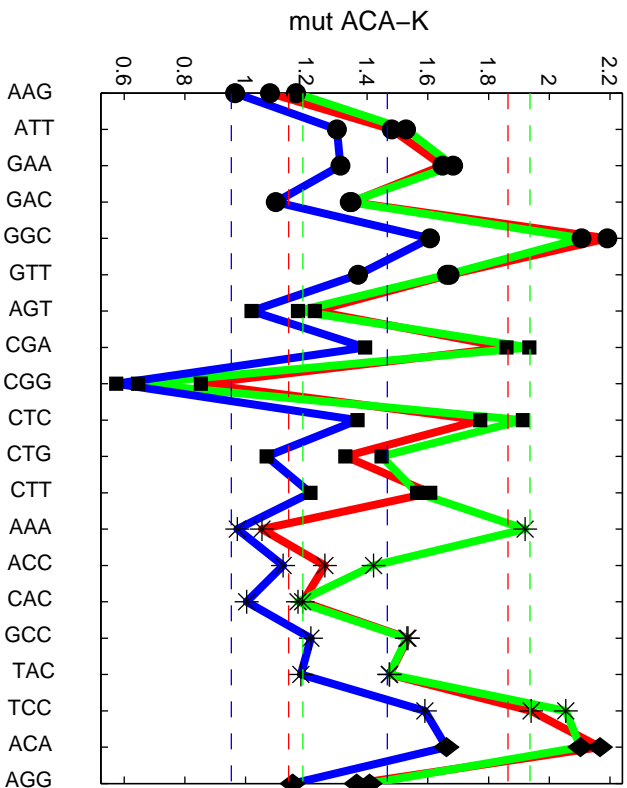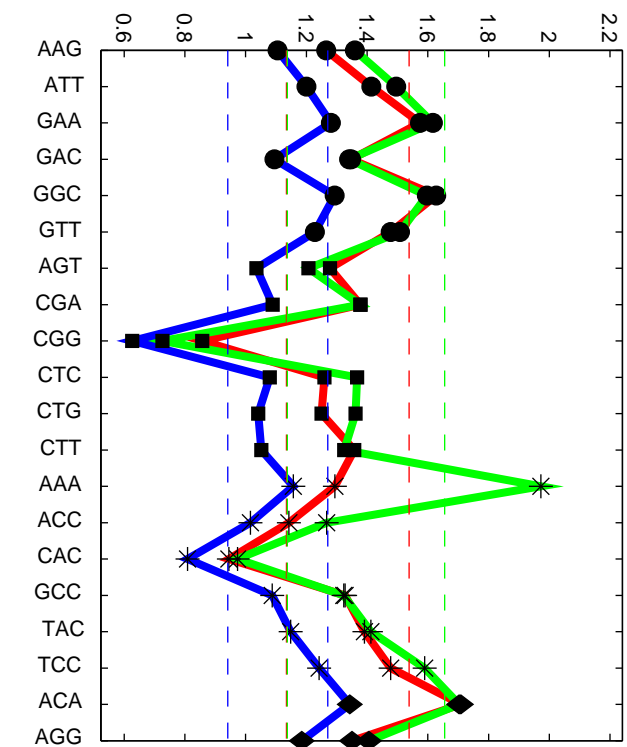

Supplement: Supplementary file 4 [file msb0010-0770-sd4.pdf]

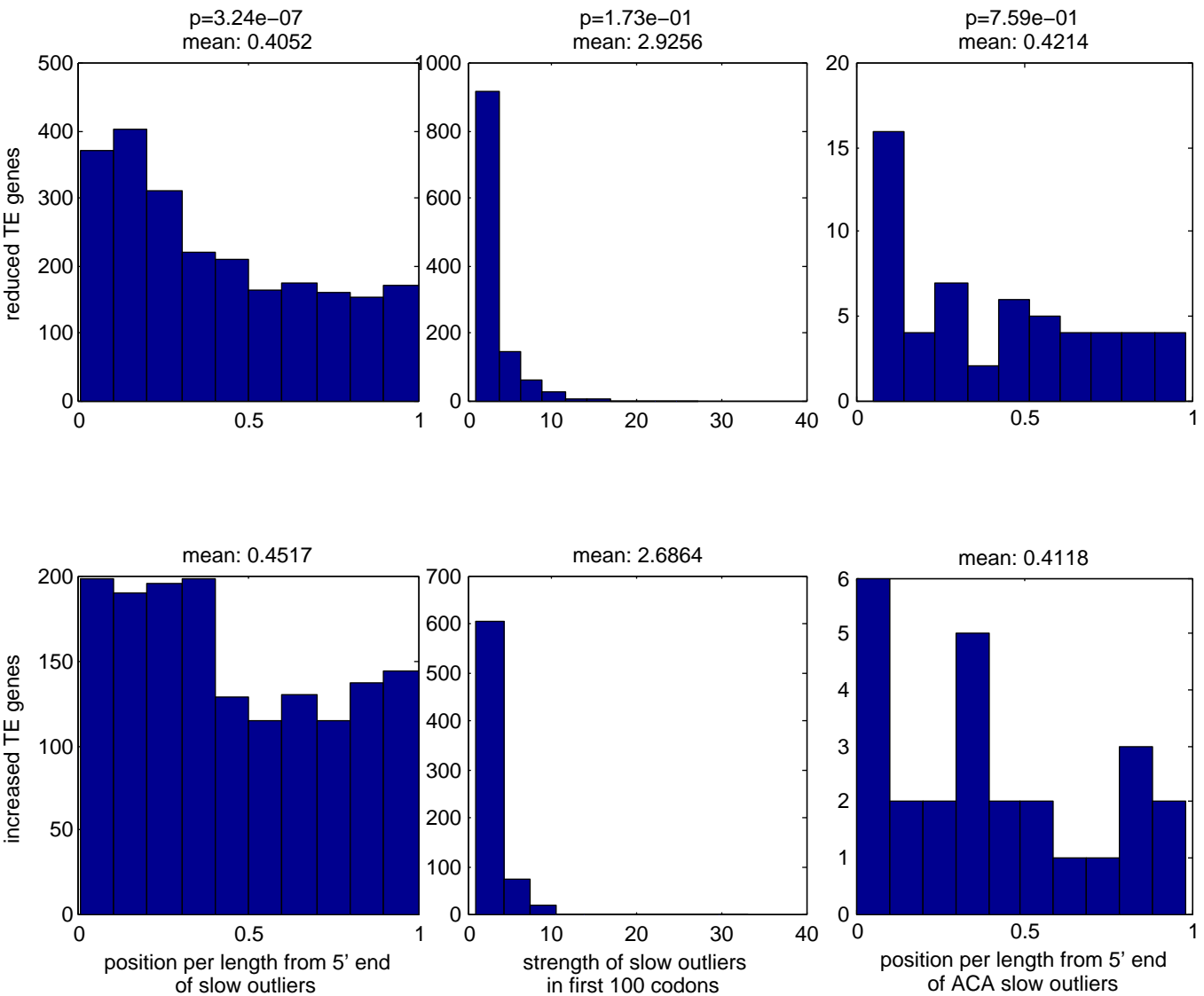

Supplement: Supplementary file 6 [file msb0010-0770-sd6.pdf]

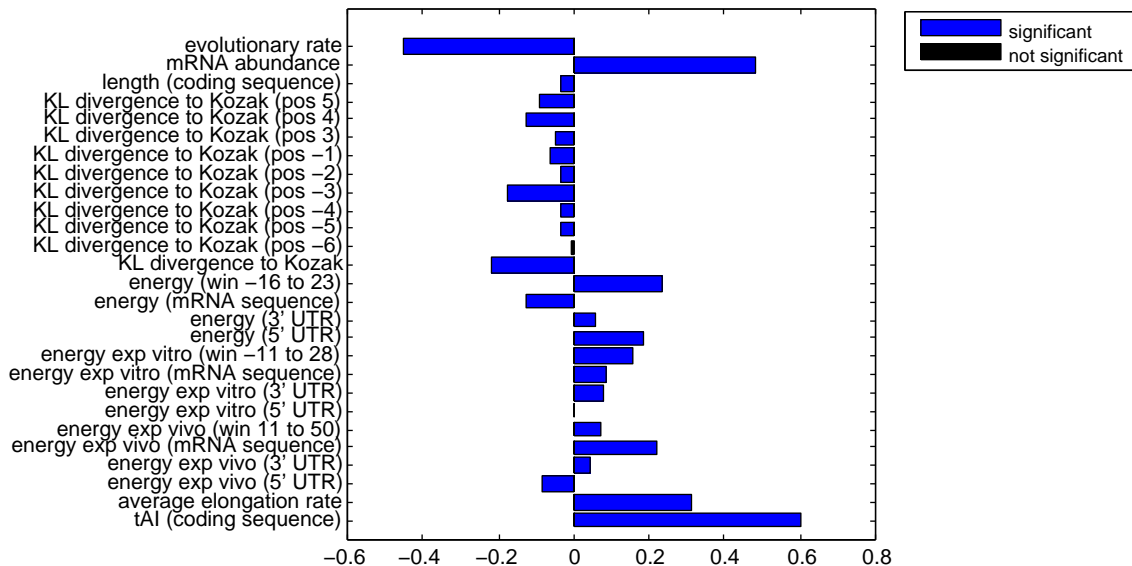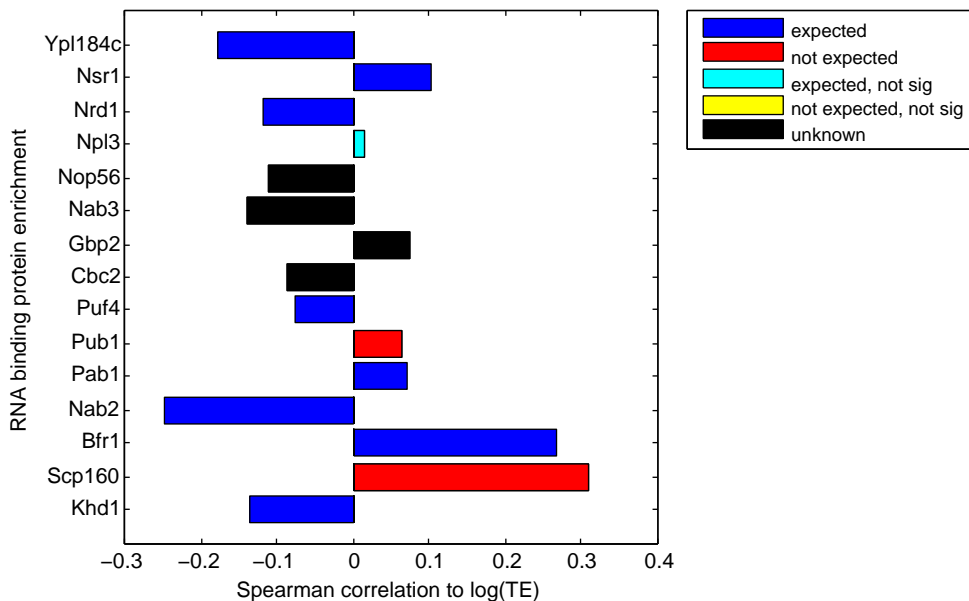

Supplement: Supplementary file 7 [file msb0010-0770-sd7.pdf]

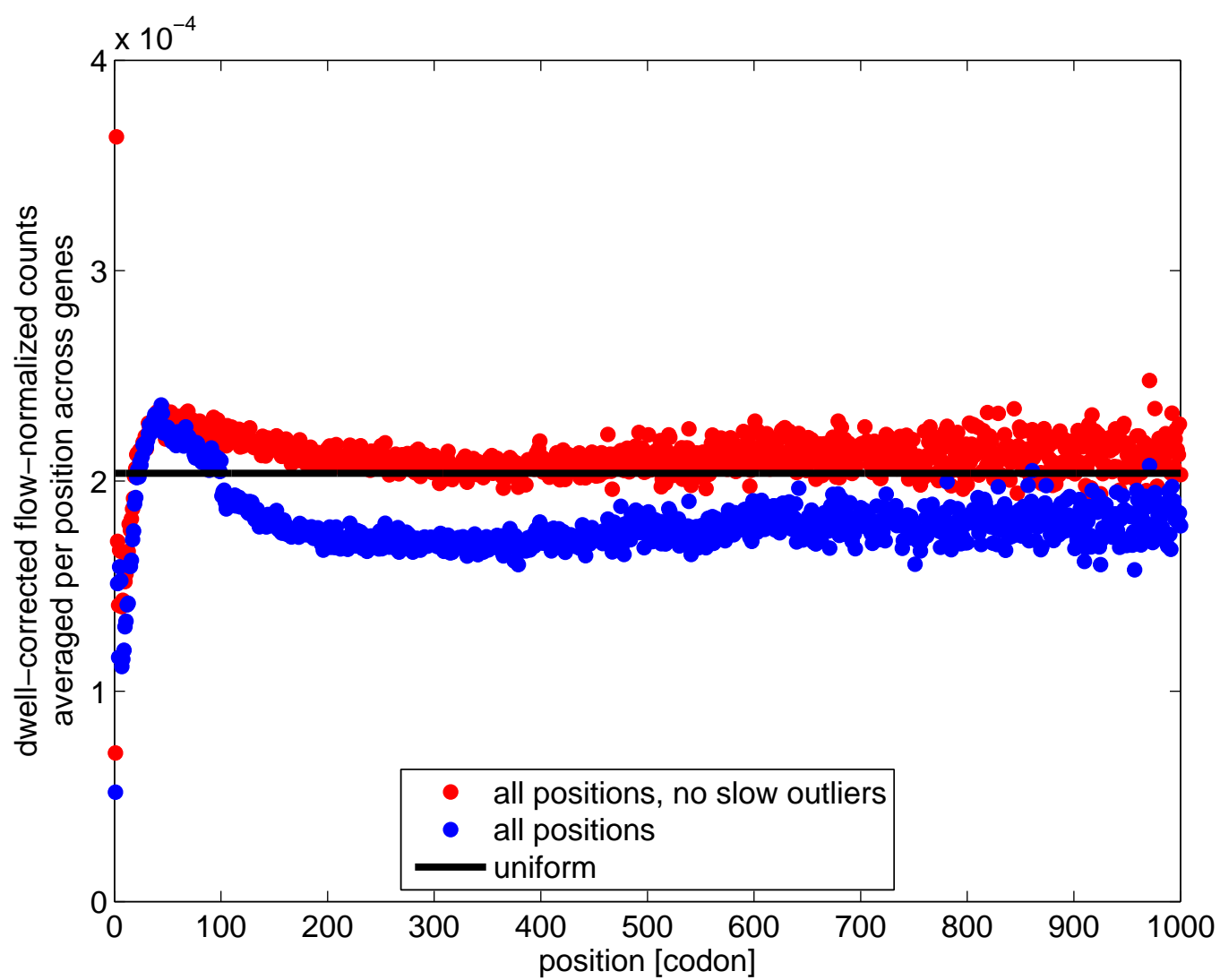

Supplement: Supplementary file 8 [file msb0010-0770-sd8.pdf]

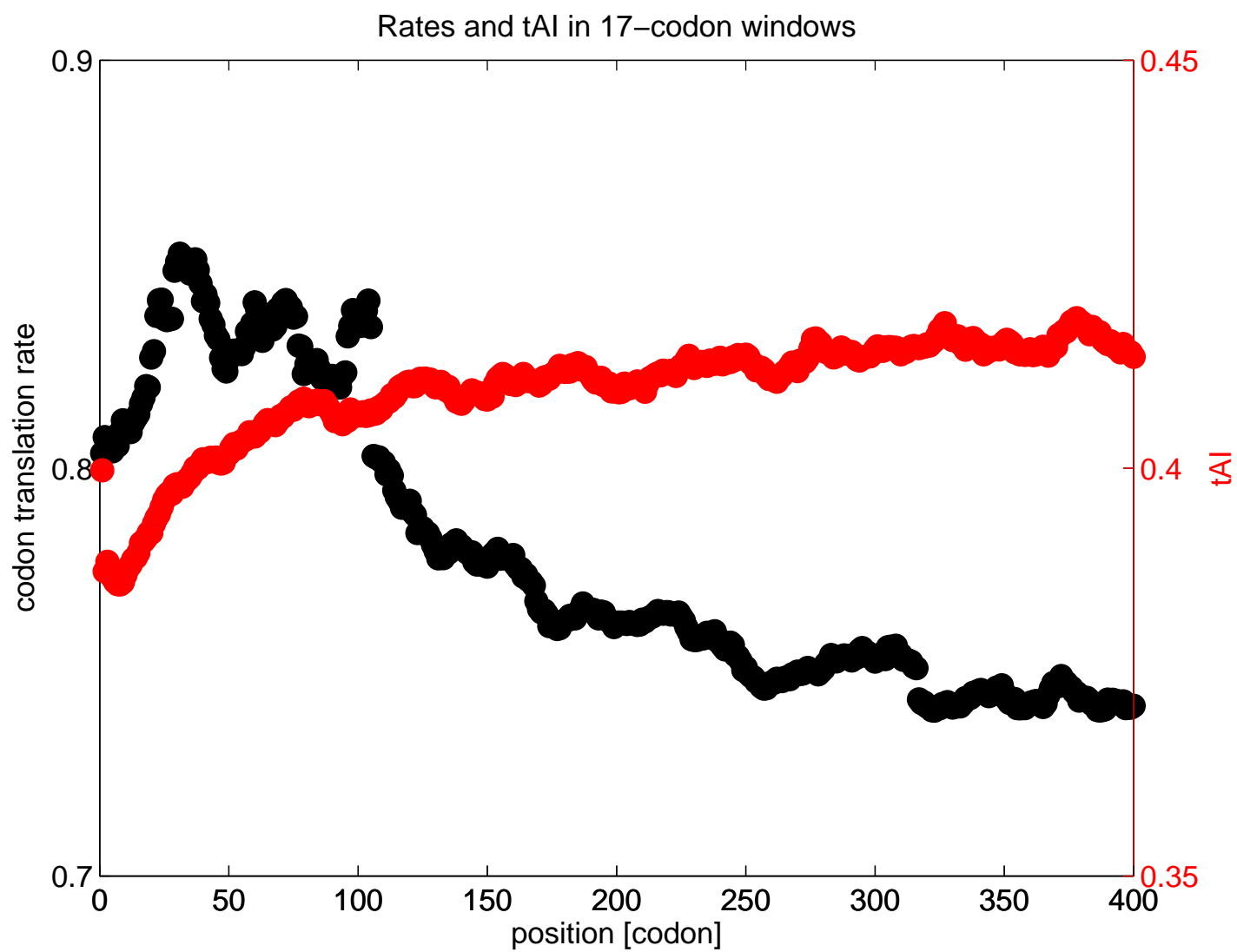

Supplement: Supplementary file 9 [file msb0010-0770-sd9.pdf]

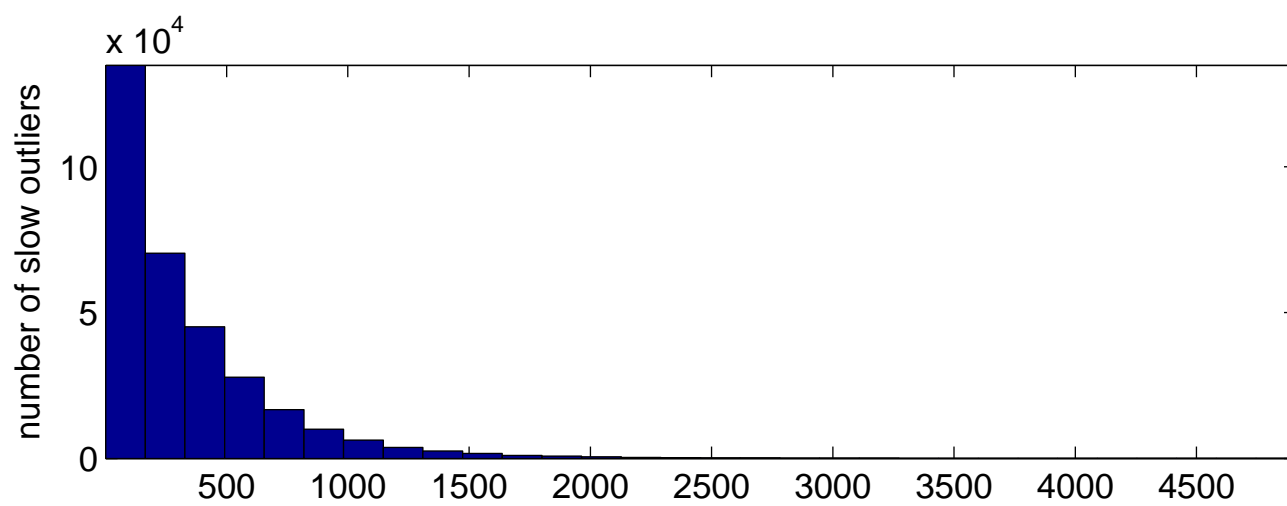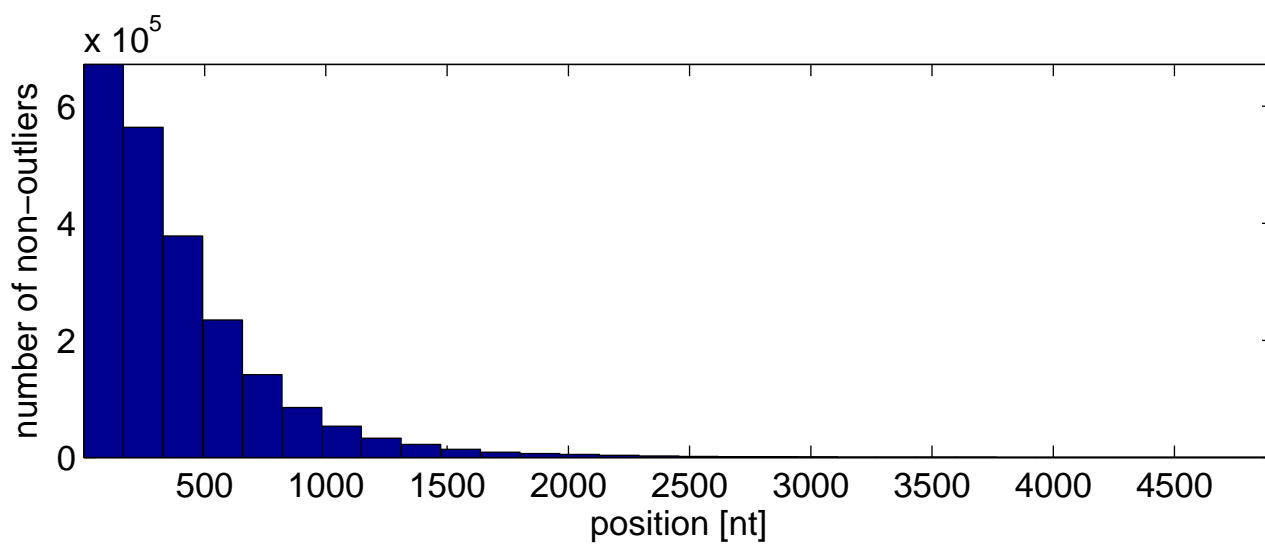

Supplement: Supplementary file 10 [file msb0010-0770-sd10.pdf]

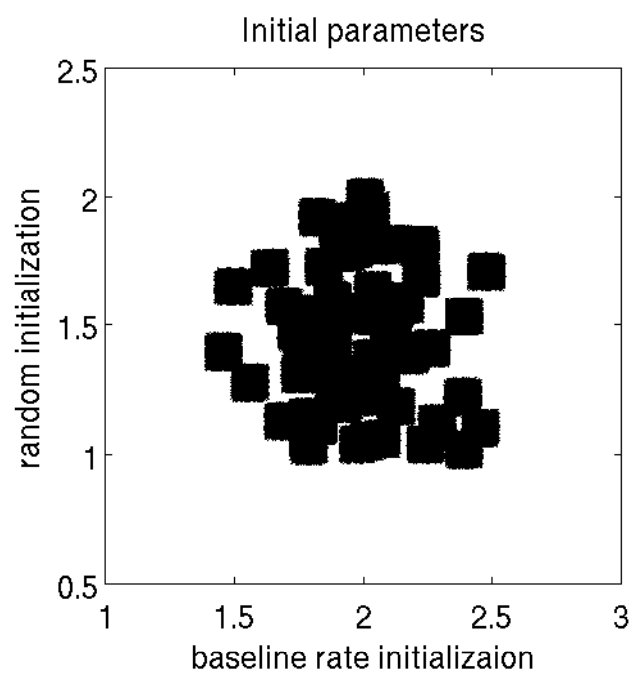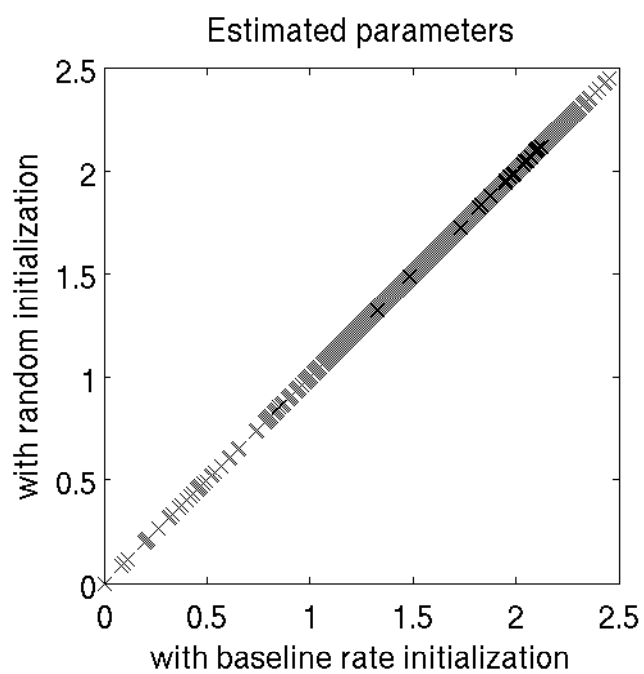

Supplement: Supplementary file 11 [file msb0010-0770-sd11.pdf]
