# Supplementary material for: Causal signals between codon bias, mRNA structure, and the efficiency of translation and elongation
Source: Mol Syst Biol. 2014 Dec 23;10(12):770. doi: 10.15252/msb.20145524 (PMC4300493; doi:10.15252/msb.20145524)
Supplement: Supplementary file 12 [file msb0010-0770-sd12.docx]

**Table S1**

Counts of tRNA in RPM (number of reads per million) in ACA-K and wild-type. The threonine tRNA recognizing the ACA codon (highlighted) is reduced to 1/3 of the wild-type level.

| **tRNA gene name (anticodon)** | **RPM (ACA-K)** | **RPM (wt)** | **RPM (ACA-K) / RPM (wt)** |
| --- | --- | --- | --- |
| tK(UUU)D | 78 | 80 | 0.98 |
| tY(GUA)F1 | 19 | 16 | 1.19 |
| tM(CAU)C | 11 | 11 | 1.00 |
| tD(GUC)B | 218 | 251 | 0.87 |
| tE(UUC)B | 582 | 428 | 1.36 |
| tN(GUU)C | 225 | 166 | 1.36 |
| tS(UGA)P | 122 | 148 | 0.82 |
| tP(AGG)N | 24 | 27 | 0.89 |
| tC(GCA)B | 82 | 58 | 1.41 |
| tQ(UUG)B | 103 | 106 | 0.97 |
| tW(CCA)G1 | 35 | 44 | 0.80 |
| tG(UCC)O | 143 | 96 | 1.49 |
| tT(UGU)G1 | 25 | 75 | 0.33 |
| tR(UCU)E | 138 | 172 | 0.80 |
| tA(AGC)D | 72 | 42 | 1.71 |
| tT(CGU)K | 9 | 9 | 1.00 |
| tV(AAC)E1 | 129 | 82 | 1.57 |
| tQ(CUG)M | 166 | 138 | 1.20 |
| tA(UGC)Q | 3 | 3 | 1.00 |
| tL(UAA)J | 72 | 81 | 0.89 |
| tI(AAU)B | 98 | 46 | 2.13 |
| tH(GUG)E1 | 328 | 266 | 1.23 |
| tT(AGU)B | 152 | 141 | 1.08 |
| tF(GAA)B | 124 | 115 | 1.08 |
| tK(CUU)C | 1328 | 1914 | 0.69 |
