# Supplementary material for: Causal signals between codon bias, mRNA structure, and the efficiency of translation and elongation
Source: Mol Syst Biol. 2014 Dec 23;10(12):770. doi: 10.15252/msb.20145524 (PMC4300493; doi:10.15252/msb.20145524)
Supplement: Supplementary file 13 [file msb0010-0770-sd13.docx]

**Table S2**

Eight categories of potential correlates to outlier strength.

| **Category** | **Features** |
| --- | --- |
| Position | Distance from 5’ end (pos)  Distance from 5’ end per length (pos-per-len)  Distance from 3’ end (pos-from-end) |
| Structure in 25nt window 15nt downstream of active  (unless indicated, features are derived from computationally-predicted structure) | Minimum free energy (energy-down)  Experimentally-derived in vitro energy (vitroDMS-energy-down) (Rouskin et al, 2013)  Experimentally-derived in vivo energy (vivoDMS-energy-down) (Rouskin et al, 2013)  Experimentally-derived in vitro inverse-energy (PARS-invenergy-down) (Kertesz et al, 2010)  Number of hairpins (hairpins-down)  Number of internal loops (internal-down)  Number of multi-loops (multi-down)  Number of stems (stems-down15)  Number of GC pairs in stems (stemsGC-down15)  Number of stems 12nt downstream (stems-down12)  Number of stems 9nt downstream (stems-down9) |
| Protein folding | Active site is inside a protein domain (is-in-domain)  End of protein domain is 30 codons upstream of active (is-end-domain-up-30) |
| Wobble bases at P-site | Is wobble base (is-wobble) |
| Reuse of tRNAs | Distance from same codon upstream (dist-prev-codon)  Distance from codon with iso-accepting tRNA upstream (dist-prev-trna)  Is same codon in 10-codon window upstream (is-prev-codon-close)  Is codon with iso-accepting tRNA in 10-codon window upstream (is-prev-trna-close) |
| KL divergence to RNA binding motifs (Brown et al. 2009) in 3-codon window 5 codons downstream of active | KL divergence over motifs and positions combined via mean (rbp-mean)  KL divergence over motifs and positions combined via min (rbp-min) |
| Nascent peptide | Charge of active codon (charge)  Mean charge in 10-codon window ending upstream of active (cluster-charge-up-1)  Fraction of Arg or Lys in 10-codon window ending upstream of active (cluster-ArgLys-up-1)  Fraction of Pro in the P and E sites (pair-Pro-up)  Fraction of Pro in two codons downstream of active (pair-Pro-down) |
| Global | Length (len)  Abundance (abund) |
