# Supplementary material for: Causal signals between codon bias, mRNA structure, and the efficiency of translation and elongation
Source: Mol Syst Biol. 2014 Dec 23;10(12):770. doi: 10.15252/msb.20145524 (PMC4300493; doi:10.15252/msb.20145524)
Supplement: Supplementary file 15 [file msb0010-0770-sd15.docx]

**Table S4**

Performance of TE regression model (see **Materials and Methods**). Error (should be low) and correlation (should be high) between predicted and actual TE is measured on 100 random test sets of genes not used during model training. Performance drops in a null model learned on randomized TE labels (last column). Performance also drops when using the original Kozak motif (middle column). Error on the training set is included to show that our model generalizes to genes not used in training (it is close to test set error).

|  | **Regression** | | **Regression (with original Kozak)** | | **Null Model** | |
| --- | --- | --- | --- | --- | --- | --- |
|  | ***Mean*** | ***Std*** | ***Mean*** | ***Std*** | ***Mean*** | ***Std*** |
| *Error* | 0.7549 | 0.0508 | 0.8443 | 0.0486 | 0.9674 | 0.0581 |
| *Error (Train)* | 0.7499 | 0.0057 | 0.8438 | 0.0051 | 0.9569 | 0.0066 |
| *Spearman r* | 0.6614 | 0.0278 | 0.5161 | 0.0382 | 0.0385 | 0.0491 |
| *Spearman p* | 0.0000 | 0.0000 | 0.0000 | 0.0000 | 0.4325 | 0.3022 |
| *Pearson r* | 0.6224 | 0.0329 | 0.5094 | 0.0381 | 0.0307 | 0.0483 |
| *Pearson p* | 0.0000 | 0.0000 | 0.0000 | 0.0000 | 0.4587 | 0.3028 |
