# Supplementary material for: Causal signals between codon bias, mRNA structure, and the efficiency of translation and elongation
Source: Mol Syst Biol. 2014 Dec 23;10(12):770. doi: 10.15252/msb.20145524 (PMC4300493; doi:10.15252/msb.20145524)
Supplement: Supplementary file 16 [file msb0010-0770-sd16.docx]

**Table S5**

Summary of main results for model variations. The first five columns are models with different constants for the second term in the objective function and the last column is a model without μ*_m_^c^* parameters (see **Materials and Methods**). Rows 1-3 represent correlation between our parameters in our model and in the model variation. Rows 4-6 represent correlation between codon translation rates in model variations and codon bias measures. Rows 7-8 represent correlation between protein synthesis rates in model variation and protein abundance measures. Results are similar to the ones reported for the model used throughout the paper (const = 100).

| **Result** |  | **const =**  **1** | **const = 10** | **const = 1000** | **const = 10000** | **const = 100000** | **No μ*_m_^c^*** |
| --- | --- | --- | --- | --- | --- | --- | --- |
| **μ*^c^* (const=100)** | r | 1.000 | 1.000 | 1.000 | 1.000 | 1.000 | 1.000 |
|  | p | 1E-202 | 5E-206 | 9E-150 | 1E-105 | 6E-95 | 1E-96 |
| **μ*_m_^c^* (const=100)** | r | 1.000 | 1.000 | 1.000 | 0.983 | 0.838 | NA |
|  | p | 0 | 0 | 0 | 0 | 0 | NA |
| **J*_m_* (const=100)** | r | 1.000 | 1.000 | 1.000 | 1.000 | 0.999 | 0.994 |
|  | p | 0 | 0 | 0 | 0 | 0 | 0 |
| **tAI** | r | 0.210 | 0.210 | 0.210 | 0.213 | 0.217 | 0.211 |
|  | p | 0.104 | 0.104 | 0.104 | 0.100 | 0.094 | 0.103 |
| **tRNA abund (Cy5)** | r | 0.144 | 0.144 | 0.140 | 0.140 | 0.140 | 0.133 |
|  | p | 0.380 | 0.4380 | 0.393 | 0.393 | 0.393 | 0.420 |
| **tRNA abund (Cy3)** | r | 0.144 | 0.144 | 0.140 | 0.140 | 0.140 | 0.133 |
|  | p | 0.417 | 0.417 | 0.429 | 0.429 | 0.429 | 0.456 |
| **PA (Newman et al)** | r | 0.7885 | 0.7885 | 0.7886 | 0.7889 | 0.7882 | 0.7782 |
| **PA (de Godoy et al)** | r | 0.6802 | 0.6802 | 0.6802 | 0.6802 | 0.6786 | 0.6710 |
