# Supplementary material for: Causal signals between codon bias, mRNA structure, and the efficiency of translation and elongation
Source: Mol Syst Biol. 2014 Dec 23;10(12):770. doi: 10.15252/msb.20145524 (PMC4300493; doi:10.15252/msb.20145524)
Supplement: Supplementary file 17 [file msb0010-0770-sd17.docx]

**Note S1**

Feature Calculations for Outlier Analysis

Computationally predicted mRNA secondary structures and associated energies were computed using Unafold v3.6 (Markham & Zuker, 2008) with the default settings. In the outlier analysis (feature “energy-down”), we ignored downstream regions with energies of 0 and above. Structural features (e.g. stems) were counted based on the structure of the whole mRNA strand, including characterized UTRs (Nagalakshmi et al, 2008). Genes without a characterized UTR were ignored for all energy-related features. For experimentally derived structure from the PARS method (Kertesz et al, 2010), we used the PARS score; genes without a PARS score were ignored.

Protein domain boundaries were based on Pfam-A domains from Pfam (Finn et al, 2008). Wobble codons were set to be those with mismatches to the anticodon and those with an “I” base in the tRNA that can recognize either a C or a U.

For RNA binding protein enrichment features in the outlier analysis, we computed the Kullback-Leibler (KL) divergence between each of the 60 motifs from Table S4 in Hogan et al (2008) and positions along each coding sequence. We then calculated the mean/minimum KL divergence in 3-codon windows 5 codons downstream of the active site and took the mean/minimum score over all motifs.

Feature Calculations for Translation Efficiency

Evolutionary rate is adjusted dN/dS from Wall et al (2005). The Kozak site motif is from Hamilton et al (1987); we ignored this in genes without characterized UTRs. Energies are calculated as described in the outlier analysis section above. Energies near the start codon are those with the most significant Spearman correlation (as calculated by looking at global maximums in spans of 20nt and taking the first such maximum). These energies are corrected for multiple hypothesis testing as described in the sliding window energy analysis. The tAI per gene or per window is the weighted average of all codons in that range, excluding stop codons.

The RNA binding protein enrichment features are the scores reported from the Significance Analysis of Microarray algorithm in Dataset S3 of Hogan et al (2008). We selected the top fifteen RBPs with the largest number of RNA targets from Table S2. Suggested “true” correlations between RNA binding proteins enrichment and translation efficiency are drawn from ribosome occupancy correlations using polysome profiling (Table S3 in Hogan et al, 2008), where possible. In other cases, we use additional literature: Puf4 is most commonly studied in mRNA stability and localization and is also likely a player in translation regulation (Goldstrohm et al, 2006). As noted in the main text, scp160 has an additional contradictory source indicating a positive role in translational efficiency (Hirschmann et al, 2014). Ypl184c was proposed to repress translation due to its association with Pab1 and mRNAs under translational control (Hogan et al, 2008). The proteins Cbc2, Gbp2, Nab3, and Nop56 do not seem to have documented direct associations with translation.

References

Finn RD, Tate J, Mistry J, Coggill PC, Sammut SJ, Hotz HR, Ceric G, Forslund K, Eddy SR, Sonnhammer EL, Bateman A (2008). The Pfam protein families database. *Nucleic Acids Res* 32: D281-8

Goldstrohm AC, Hook BA, Seay DJ, Wickens M (2006) PUF proteins bind Pop2p to regulate messenger RNAs. *Nat Struct Mol Biol* 13: 533-39

Hirschmann WD, Westendorf H, Mayer A, Cannarozzi G, Patrick C, Jansen R-P (2014) Scp160p is required for translational efficiency of codon-optimized mRNAs in yeast. *Nucleic Acids Res* 42(6): 4043-55

Hogan DJ, Riordan DP, Gerber AP, Herschlag D, Brown PO (2008) Diverse RNA-Binding Proteins Interact with Functionally Related Sets of RNAs Suggesting an Extensive Regulatory System. *PLoS Biol* 6(10): e255

Markham NR & Zuker M (2008) UNAFold: Software for Nucleic Acid Folding and Hybridization. *Methods in Mol Biol* 453: 3–31

Wall DP, Hirsh AE, Fraser HB, Kumm J, Giaever G, Eisen MB, Feldman MW (2005) Functional genomic analysis of the rates of protein evoluation. *Proc Natl Acad Sci* 15(102): 5483-8
