# Supplementary material for: Causal signals between codon bias, mRNA structure, and the efficiency of translation and elongation
Source: Mol Syst Biol. 2014 Dec 23;10(12):770. doi: 10.15252/msb.20145524 (PMC4300493; doi:10.15252/msb.20145524)
Supplement: Supplementary file 18 [file msb0010-0770-sd18.docx]

Supplementary Legends

**Figure S1**

Correlation between experimental measures of protein abundance – de Godoy et al (2008) and Newman et al (2006) – and estimated flow. As a baseline, we compare against protein abundance calculated as the average footprint count per gene.

**Figure S2**

Overexpression of tRNA^Arg(CCU)^ does not significantly alter amino acid charging levels. Bulk RNAs from strains as indicated were resolved at pH 5 by PAGE, transferred, and hybridized with oligonucleotide probes specific for tRNA species as indicated, and relative tRNA^Arg(CCU)^ levels and charging levels were evaluated as described in **Materials and Methods**. Solid arrows show deacylated tRNAs; dashed arrows show charged tRNAs; % charged refers to tRNA^Arg(CCU)^.

**Figure S3**

The ratio between estimated mutant and wild-type rates. The mean (solid black line) and standard deviation (dashed line) are shown. ACA-K has a larger spread, but the manipulated codon (shown in red) is not an outlier in any sample. Codons are grouped and sorted by amino acid.

**Figure S4**

The ratio of mutant to wild-type footprint count per codon, averaged over the first 5 occurrences of the codon per gene over all genes, presented for the three mutant samples. Counts are normalized by the average in the 15-codon window before (red line), after (green line), or around (blue line) the codon. We show a subset of the codons: the 5 with lowest tAI (dots), the 5 with highest tAI (squares), and the 6 with middle tAI (stars), in addition to the two codons ACA and AGG (diamonds). In each case, if the manipulated codon of interest induces a change in speed under the common hypothesis (lower for ACA-K and higher for AGG-OE and AGG-QC), we expect a corresponding peak or valley, respectively, in the presented ratio. However, the ratios at ACA and AGG are not significantly higher than 1-standard deviation (dotted line) or than the other representative codons.

Left: Counts are raw footprint counts.

Right: Counts are dwell-corrected footprint counts.

**Figure S5**

The analysis of Figure 4 repeated on flow instead of TE. As before, wild-type and mutant flows generally agree. Correlations between the ratio of mutant flow to wild-type flow and the percent of codon per gene are not higher for the manipulated codons compared to other codons, despite the dramatic change in tRNA abundance.

**Figure S6**

Distribution of three features among reduced TE genes and increased TE genes in ACA-K. Distributions are skewed for reduced TE genes (with lower TE in mutant compared to wild-type) toward initiation signals that could confound the TE decrease. Slower-than-expected codons with an excess number of ribosome counts are defined formally as “outliers” (see **Materials and Methods**). Each feature distribution is calculated over all positions in the genes in the specified gene set (either reduced TE genes or increased TE genes) satisfying the specified criteria (a position that is a slow outlier, a position that is a slow outlier in the first 100 codons, or a position that is a slow outlier and an ACA codon). The feature distributions for reduced TE versus increased TE genes are distinct (p-values shown are calculated to be significant under a Kolmogorov-Smirnov test). Outlier positions are calculated in the ACA-K mutant.

**Figure S7**

Correlation between log(TE) and gene-level features, including *cis*-features and RNA binding protein enrichment (see **Materials and Methods**). Significant threshold is p = 0.05. (See Supplementary Note S1 for how expected correlations for the RNA binding proteins were determined.)

**Figure S8**

Dwell-corrected footprint counts normalized by flow, geometrically averaged per position over all genes aligned by start codon (ignoring 0 footprint counts). Removing slow outliers (red curve) reduces the peak in density at ~44 codons (132 nt).

**Figure S9**

The tAI in sliding windows of 17-codons averaged across all the genes aligned by start codon (red curve). The same analysis with our estimated codon translation rates (scaled up by 1000) (black curve) show that rates at the 5’ end are not lower compared to the rest of the gene.

**Figure S10**

Histograms of positions of slow outliers and non-outliers are similar.

**Figure S11**

Two different initializations of the parameters for the translation model yield estimated parameters that are nearly exact. This demonstrates the model is robust to initialization.

**Table S1**

Counts of tRNA in RPM (number of reads per million) in ACA-K and wild-type. The threonine tRNA recognizing the ACA codon (highlighted) is reduced to 1/3 of the wild-type level.

**Table S2**

Eight categories of potential correlates to outlier strength.

**Table S3**

Correlation between outlier strength and features. Significant ones (see **Materials and Methods**) are highlighted. The first two rows per feature correspond to Pearson correlation; the last two are Spearman. (See Supplementary Note S1 for more discussion of the correlations.)

**Table S4**

Performance of TE regression model (see **Materials and Methods**). Error (should be low) and correlation (should be high) between predicted and actual TE is measured on 100 random test sets of genes not used during model training. Performance drops in a null model learned on randomized TE labels (last column). Performance also drops when using the original Kozak motif (middle column). Error on the training set is included to show that our model generalizes to genes not used in training (it is close to test set error).

**Table S5**

Summary of main results for model variations. The first five columns are models with different constants for the second term in the objective function and the last column is a model without μ*_m_^c^* parameters (see **Materials and Methods**). Rows 1-3 represent correlation between our parameters in our model and in the model variation. Rows 4-6 represent correlation between codon translation rates in model variations and codon bias measures. Rows 7-8 represent correlation between protein synthesis rates in model variation and protein abundance measures. Results are similar to the ones reported for the model used throughout the paper (const = 100).
