# Supplementary material for: Causal signals between codon bias, mRNA structure, and the efficiency of translation and elongation
Source: Mol Syst Biol. 2014 Dec 23;10(12):770. doi: 10.15252/msb.20145524 (PMC4300493; doi:10.15252/msb.20145524)
Supplement: Supplementary file 5 [file msb0010-0770-sd5.pdf]

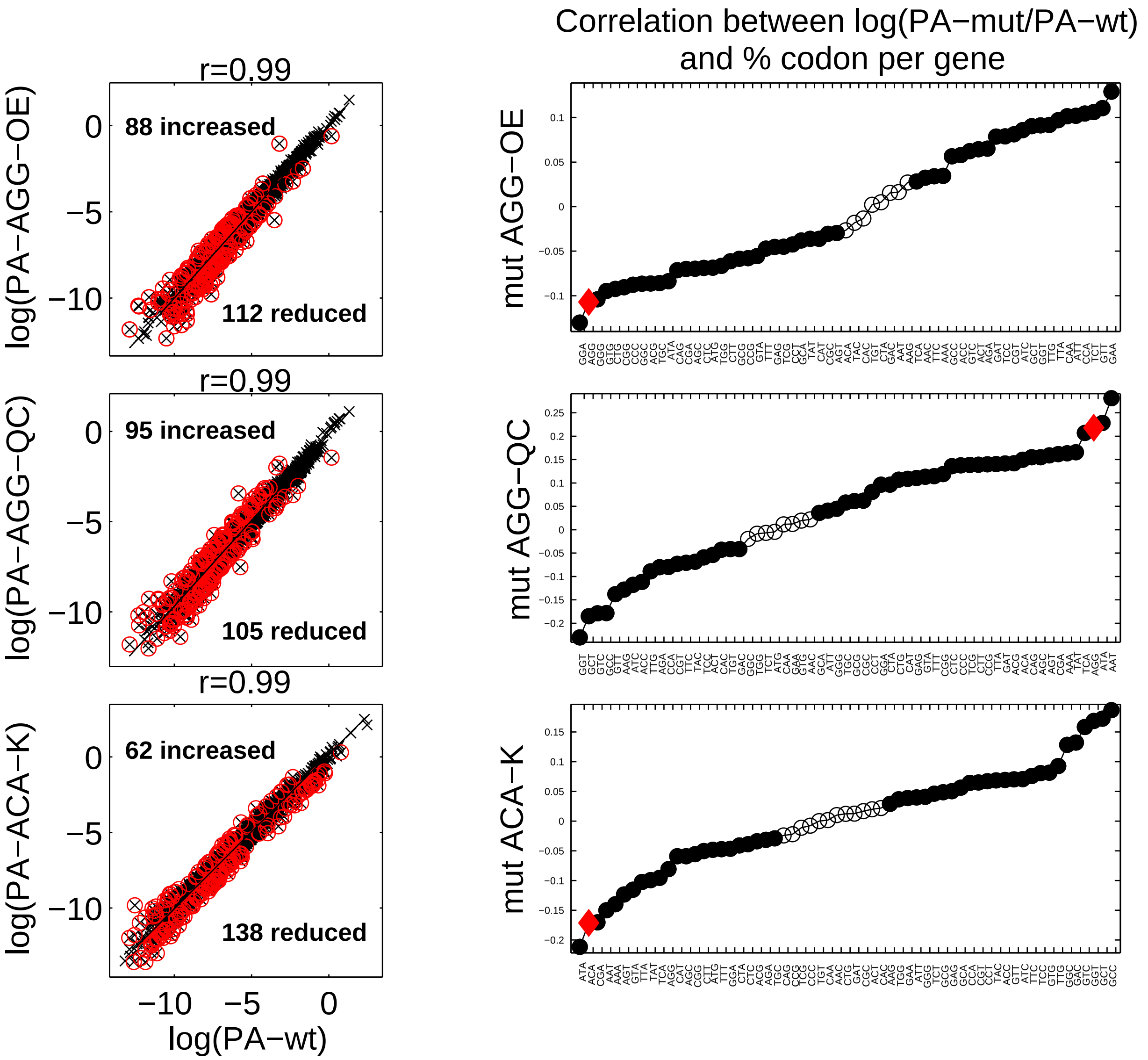

Correlation between log(PA−mut/PA−wt)
and % codon per gene
r=0.99
88 increased
112 reduced
log(PA−AGG−OE)
r=0.99
95 increased
105 reduced
log(PA−AGG−QC)
r=0.99
62 increased
138 reduced
log(PA−ACA−K)
log(PA−wt)
mut AGG−OE
mut AGG−QC
mut ACA−K
