# Supplementary material for: Causal signals between codon bias, mRNA structure, and the efficiency of translation and elongation
Source: Mol Syst Biol. 2014 Dec 23;10(12):770. doi: 10.15252/msb.20145524 (PMC4300493; doi:10.15252/msb.20145524)
Supplement: Supplementary file 20 [file msb0010-0770-sd20.pdf]

Causal signals between codon bias, mRNA structure and the efficiency of translation and elongation

Cristina Pop, Silvi Rouskin, Nicholas T. Ingolia, Lu Han, Eric M. Phizicky, Jonathan S. Weissman, Daphne Koller

Corresponding author: Cristina Pop, Stanford University

Review timeline:

Submission date:	23 June 2014
Editorial Decision:	06 August 2014
Revision received:	05 November 2014
Editorial Decision:	20 November 2014
Revision received:	02 December 2014
Accepted:	03 December 2014

Editor: Maria Polychronidou

Transaction Report:

(Note: With the exception of the correction of typographical or spelling errors that could be a source of ambiguity, letters and reports are not edited. The original formatting of letters and referee reports may not be reflected in this compilation.)

1st Editorial Decision 06 August 2014

Thank you again for submitting your work to Molecular Systems Biology. We have now heard back from the three referees who agreed to evaluate your manuscript. As you will see from the reports below, the referees acknowledge that, while the presented conclusions are not entirely novel, they are drawn based on carefully designed computational and experimental analyses and represent a potentially useful contribution to the field. However, they raise a series of concerns, which should be carefully addressed in a revision of the manuscript.

Without repeating all the comments listed below, some of the more fundamental issues are the following:
- Reviewer #1 points out that additional experimentation is required to more convincingly support the lack of effect of tRNA over-expression and tRNA-body exchange on elongation rates.
- Reviewer #2 refers to the need to demonstrate that the use of the term constraining dwell times to be similar across the transcriptome, does not affect the resulting conclusions.
- The advantages of the presented computational approach should be discussed and the overall findings and conclusions need to be better placed in the context of the current knowledge.

Reviewer #1:

In this manuscript, Pop et al. describe ribosome profiling experiments they performed in the yeast Saccharomyces cereivisiae to study the effects of tRNA abundance on codon elongation rate. The approach involves overexpression, deletion, or body exchange in tRNA genes, followed by ribosome profiling and explicit modelling of parameters relevant to translation, such as TE(m)

(translation efficiency, or flow of ribosomes per mRNA molecule), and mu(c) (codon-specific elongation rates). Surprisingly, the authors find that neither tRNA overexpression nor tRNA depletion influence the dwell time of the ribosome at the expected codon. Most other conclusions are the same as in previous ribosome profiling studies, including the lack of correlation between codon adaptation and codon elongation rate, or a negative effect of RNA secondary structure near the start codon on translation efficiency.

Although this is a well-designed study, the main conclusions are so surprising as to make them questionable. It is unclear why the increase or decrease of active tRNA levels would not lead to a corresponding change in codon elongation rates. The problem could be experimental (eg. tRNA overexpression), or computational (eg. identification of A-site codon). In any case, the authors should make an effort to identify the factors that could have prevented the finding of a relationship between tRNA level and elongation rate, and eliminate such factors. If a relationship between tRNA level and elongation rate cannot be found despite sincere efforts, then the authors should propose a theoretical explanation of such a lack of correlation.

Specific comments:

A simple explanation for the lack of effect of tRNA overexpression on codon elongation rates is that tRNA overexpression did not result in increased production of aminoacylated tRNA or ternary complex. This needs to be tested experimentally before the tRNA overexpression can be interpreted. Similarly, the exchange of tRNA body for a "faster" one might not have increased the "speed" of the tRNA - experiments need to be done to support the expected effect of body swap on tRNA speed.

AGG tRNA overexpression (or body replacement) does not seem to affect AGG codon dwell time, but it seems to influence dwell times at CGA and CGG codons. Why would that be?

Although the computational model sounds theoretically appealing, in practice simple footprint counts predict protein abundance just as well as the model does (Fig E1). The practical advantages of the method (if any) should be better justified. The method also needs to be discussed in the context of previous methods used to analyse ribosome profiling data.

The model assumes that codon-specific elongation rates mu(mc) systematically differ between transcripts. What happens when mu(mc) is set to be equal to mu(c) for all transcripts?

In the abstract, the authors suggest that codon bias arises from selection for rapidly elongated codons, but in the results section, they show that high-tAI codons are not elongated more rapidly than others. So are high-tAI codons rapidly elongated or not? The manuscript should present an internally consistent view, or at least acknowledge the discrepancy.

In Table E3, the authors should show the mean +/- SD of sequence-derived correlates, calculated separately for slow outliers, non-outliers, and fast outliers. This would help estimate the magnitude of the various effects. These findings are potentially interesting and should be discussed in more detail.

The analysis of covariates of translation efficiency is potentially interesting, but not presented adequately. Figure E6 should be expanded to include the other covariates tested, included in the main text, and discussed in more detail. The correlations with RNA-binding protein enrichment are interesting, but not at all discussed.

The sentence "However, we observe a correlation between position and slow outlier strength even when separating outliers by codon (Figure 5)" and Figure 5 itself are unclear.

Page 23, ribosome fragments -> ribosome-protected fragments

In table E1, are these counts of tRNA genes, or tRNA molecules per cell, or some other unit?

The meaning of figure E5 is unclear. for example, what are "fast outlier genes" and "position per length of ACA slow outliers" in the bottom right panel?

© European Molecular Biology Organization

Reviewer #2:

This is a comprehensive study that deals with multiple potential effects of genes' sequence on translation efficiency (TE). The paper carefully examines various factors that could affect TE, including codon usage, the tRNA adaptation index (including effective manipulations of the tRNA pool itself), mRNA secondary structure etc.
They conclude that mainly the secondary structure, more than anything else affect TE. The lack of any effect of tRNA manipulation on TE is surprising.
The paper is very well written, the work was done at the highest standard, both experimentally and computationally, and the conclusions are valid.
I do have two issues, though
1. Novelty - the question and the conclusion are not highly novel. Many recent studies, cited here, and beyond, have recently come to the same conclusion. The combination of tRNA deletion does represent some novelty.
2. A conceptual point is still not in place: clearly many previous studies have shown a string correlation between the tAI or CAI and protein expression level. The current lack of correlation with TE is thus hard to explain. The author mention the paper by Kudla and Plotkin (which came to same conclusion as in the current work - that secondary structure, not codon optimality affect TE), who offer a compelling reasoning to the conundrum: highly expressed genes are evolutionarily pressured to be codon optimized more than lowly expressed genes. The current authors appear to support that notion. Yet, it is still hard to grasp, how could that evolutionary effect be relevant if codon optimization had no effect on TE.
I'm very curious to read the authors' view on this, in a discussion.

Reviewer #3:

This is a very nice study that provides solid evidence, based on experimental manipulation, for role of codon usage in shaping translational efficiently. In fact it is one of the few, if not first, study that combines ribo-profiling with the experimental manipulation of tRNA abundances -- an ideal, if technically difficult, way to assess the mechanisms by which codon usage might influence elongation rates and translational efficiency more generally. The work also has the merit that it infers synthesis rate from profiling data under a simple mechanistic model, with parameters fitted (mostly) by maximum likelihood -- as opposed to the standard, somewhat ad-hoc definition of translational efficiency that exists in the literature. The overall conclusions -- that codon usage does not modulate protein synthesis rates, but rather that it is selected in highly expressed endogenous genes in order to reduce the load on translational machinery; and that synthesis rates are generally limited by the rates of translation initiation -- are not novel. Indeed, these conclusions are in line with the prevailing view in the field, which dates back to Andersson and Kurland (1990) and Bulmer (1991). And, yet, the experiments here demonstrate these results far more directly than has previously been possible, and they provide detailed subtleties for the role of structure near the Kozak sequence in yeast. The paper also strongly contravenes, with direct experimental data, the notion of Tuller (2010) that slow elongation near the 5' ends of genes is caused by the presence of "slow" codons.

Other merits of the paper include the use of DMS probing as opposed to computational prediction of mRNA structure -- something that should perhaps be mentioned in the abstract.

I have one major concern, which I feel the authors can address through some additional analyses. I also have a list of relatively minor comments that the authors can address by textual revisions.

MAJOR CONCERN

In order to estimate dwell times at all positions in the transcriptome, as well as the overall flow rate across each message, the authors optimize an expression that is not simply the likelihood of these parameters, given the data, but that also includes a regularization term forcing dwell times to be similar, across the transcriptome, for all instances of a given codon. The authors argue that this term is neccesary for technical reasons (the data are insufficient to maximize so many parameters' likelihoods; and the complete likelihood landscape would complications parameter estimation because of local maxima). Moreover, the regularization constraint also has have some intuitive basis. And, yet, there is undoubtably the possibility that the same instance of a codon will be

elongated at different rates than other instances depending upon, say, where it occurs along the length of a transcript (this is true even within the authors' own analysis) or perhaps depending upon the identity of the transcript, due to, for example, spatial segregation of transcripts within the cell, etc. The authors should be more circumspect about the use of their regularization term when inferring dwell times. I suggest the authors perform an additional analysis using an expression that modulates the regularization term by a scaling factor, beta, and then show how their qualitative results vary with beta -- in order to determine to what extent this additional term, made for technical convenience, has influenced their inferred parameters and their conclusions.

MINOR COMMENTS:

1. The word "yeast" should appear somewhere in the title or abstract, so that readers can quickly know what organism is being studied.

2. For better or worse, the term TE has already been defined in the literature as simply RPF rpkm divided by mRNA rpkm. I agree that this is not always the best measure of actual synthesis rates per mRNA molecule, and I appreciate the attempt to rigorously estimate such rates from data using a mechanistic model. But nonetheless I encourage the authors to use a different terminology for their inferred synthesis rates, Jm/Mm -- as opposed to "TE", which already has a clear and consistent definition in the literature.

3. I find Figure 5, and the entire notion of "outlier strength", difficult to interpret, despite several re-readings. Outlier strength is initially defined position by position, but it is then discussed in the context of "outlier genes". Is this simply an abuse of notation, or is different measure of "outlier" defined on a gene-by-gene basis, by summing over positions within each gene?

4. The authors could do a better job placing their results in the literature on determinants of protein synthesis. In particular, the authors write:

"Although codon usage is a strong correlate to TE (Figure E6), our mutant experiments suggest (via the correlation between codon bias and tRNA abundance) that codon usage may not causally influence efficiency. Rather, we propose that for highly translated genes, there is selection for codons that are rapidly elongated, so as to reduce overall utilization of the translation machinery. This would yield a correlation between codon usage and TE without implying that increasing codon bias optimizes efficiency on individual genes (Welch et al, 2009)."

This is perfectly reasonable conclusion given the authors' data. But as it currently stands, the manuscript implies that the authors are the first to propose this view of translation. In fact, this view of translation is very old; it was first discussed by Andersson & Kurland (1990) and Bulmer (1991), and it was more recently elucidated in virtually the same exact terms by Kudla et al 2009, who wrote:

"In one view, high CAI induces strong protein expression, whereas we argue that strong expression induces selection for high CAI... We propose that the correspondence between codon adaptation and expression level among endogenous E. coli genes arises from selection to make translation efficient at a global level, rather than at the level of individual genes. High CAI increases the elongation rate, but because initiation is rate-limiting in translation, elongation rate does not significantly affect expression. On the other hand, rapid elongation sequesters fewer ribosomes on the message, thereby increasing the total rate of protein synthesis and accelerating cell growth. A similar model for codon preference has been proposed by Andersson and Kurland"

I would suggest the authors re-write this part of their Discussion, to explain that their results support the widely held view on the direction of causality between codon bias and expression level -- a view that dates back to Andersson and Kurland (1990) and Bulmer (1991), and which has already been validated by manipulative experiments in microbes.

Likewise, the authors' statement that "our experimental results suggest a model in which initiation is rate-limiting in physiological conditions" is, again, completely in line with the standard view of translation dating back to Bulmer 1991. And so too is the conclusion that, for highly expressed genes, modulating codon usage can replenish translational machinery and thereby speed up initiation

and rates of synthesis globally in the cell. The authors might want to consider the value of emphasizing that their experiments qualitatively augment the experimental support for this view of translation, which has a long precedent in the literature.

1st Revision - authors' response 05 November 2014

Response to Reviewer #1:

1. *The authors should make an effort to identify the factors that could have prevented the finding of a relationship between tRNA level and elongation rate, and eliminate such factors. If a relationship between tRNA level and elongation rate cannot be found despite sincere efforts, then the authors should propose a theoretical explanation of such a lack of correlation.*

   A simple explanation for the lack of effect of tRNA overexpression on codon elongation rates is that tRNA overexpression did not result in increased production of aminoacylated tRNA or ternary complex. This needs to be tested experimentally before the tRNA overexpression can be interpreted. Similarly, the exchange of tRNA body for a "faster" one might not have increased the "speed" of the tRNA - experiments need to be done to support the expected effect of body swap on tRNA speed.

We thank the reviewer for the suggestion to verify that tRNA expression manipulations produce the expected effects on the abundance of charged tRNAs. We report the data on the abundance of charged AGG tRNA upon overexpression and for the body swap in a new supplement (Figure E2), which are explained in the section "Codon translation is not affected by tRNA abundance or body sequence" (p. 10) and in Materials and Methods ("Analysis of tRNA charging and relative RNA levels", p. 27). These indicate that our manipulation of tRNA copy number achieved the intended change in charged tRNA levels within the cell, and discrepancies in the amount of charged tRNA cannot explain the observed insignificant correlation.

More broadly, the causal relationship between tRNA abundance and elongation rate remains a topic of active debate (cf. Plotkin). We believe that one important impact of our work is its contribution to this open question, and we have added to our "Discussion" (paragraph 1) in order to better situate our work in this context. Briefly, our novel experimental manipulation of tRNA indicates that the weak relationship between measures of codon usage bias and elongation rate in wild-type cells is not causal; rather, our results are explained, for example, by the hypothesis that codon bias is a result of efficient use of translation machinery. The selection against disfavored codons, particularly in highly-expressed genes, in turn may force tRNA levels to decline. Our study and discussion of outliers and biological properties of the RNA aim to address the role of variable elongation rates.

2. *AGG tRNA overexpression (or body replacement) does not seem to affect AGG codon dwell time, but it seems to influence dwell times at CGA and CGG codons. Why would that be?*

We believe this effect may be related to CGA and CGG codons being amongst the bottom three codons in terms of frequency in the genome. This could cause greater variance in the raw counts and in analyses comparing raw counts, as in Figure E4. (Concretely, the frequency of CGA and CGG codons in the ratio computed in Figure E4 compared to that for AAG, for example, is 0.29 and 0.18, respectively, whereas this is on average 0.8 for the other codons and >0.5.)

3. *Although the computational model sounds theoretically appealing, in practice simple footprint counts predict protein abundance just as well as the model does (Fig E1). The practical advantages of the method (if any) should be better justified. The method also needs to be discussed in the context of previous methods used to analyse ribosome profiling data.*

Paragraph 2 in the "Discussion" originally addressed some of the advantages of our model. As suggested, we have also added additional discussion to that paragraph in order to better place our

© European Molecular Biology Organization

method among others used for ribosome profiling analysis and to highlight the comparative advantages.

4. *The model assumes that codon-specific elongation rates mu(mc) systematically differ between transcripts. What happens when mu(mc) is set to be equal to mu(c) for all transcripts?*

This is similar to increasing the constant on the regularization term in the objective function. We added to the paragraph 1 under the "Translation model" section of "Materials and Methods" a description showing that the main results are mostly agnostic under the model with only mu(c) (namely, the correlation between codon translation rate and codon bias, correlation to protein abundance, and outlier correlations), except for a decrease in correlation to protein abundance. We also added Table E5 to summarize these results. We note that even if there is no difference in codon rate between transcripts, the current model with mu(mc) would be able to infer the same rate mu(mc) across the m. However, if there is a transcript-level difference and we do not have this additional layer of parameters, we may not be able to detect it.

5. *In the abstract, the authors suggest that codon bias arises from selection for rapidly elongated codons, but in the results section, they show that high-tAI codons are not elongated more rapidly than others. So are high-tAI codons rapidly elongated or not? The manuscript should present an internally consistent view, or at least acknowledge the discrepancy.*

We thank for the reviewer for pointing out this discrepancy. We have removed references to "rapidly elongated" codons from the "Abstract" and paragraph 1 of the "Discussion". Our hypothesis is that codon bias and translation efficiency are correlated, but that codon bias does not necessarily reflect the actual elongation rate of codons. Rather, we meant to say that codon bias reflects "preferred" codons, which likely arise due to various selective pressures and could, for example, help utilize translational machinery more efficiently.

6. *In Table E3, the authors should show the mean +/- SD of sequence-derived correlates, calculated separately for slow outliers, non-outliers, and fast outliers. This would help estimate the magnitude of the various effects. These findings are potentially interesting and should be discussed in more detail.*

We have added the SD column to Table E3 for slow outliers and non-outliers (originally SD was folded into the calculation for the mean and that column was not properly labeled; we have now separated the mean and SD into two columns). As described in the "Outlier model" section of the "Materials and Methods", we believe that fast outliers do not contain as much reliable information as non-outliers. For that reason, we wish to maintain just the slow outlier and non-outlier columns in Table E3.

As suggested by the reviewer, we also added further discussion on the outliers: we moved most of the "Outlier Analysis" section of Note E1 to the main text at the end of the last paragraph in "Factors correlated with elongation efficiency". We also added a sentence about the practical use of the outlier analysis and its limitations to Paragraph 6 of the "Discussion".

Finally, the SD for "energy-down" was originally very high because we included positive energies output by the folding prediction software, which are not meaningful. So we also re-calculated the numbers in that row of Table E3 with positive energies excluded (but as expected, that feature is still insignificant and no other claims are affected).

7. *The analysis of covariates of translation efficiency is potentially interesting, but not presented adequately. Figure E6 should be expanded to include the other covariates tested, included in the main text, and discussed in more detail. The correlations with RNA-binding protein enrichment are interesting, but not at all discussed.*

We included a new paragraph at the end of the section "Factors correlating with translation

efficiency" which discusses in more detail all the covariates in Figure E7 (renumbered due to a new addition to the figure set). We had originally discussed the correlations between TE and the RNA-binding proteins briefly in Note E1 (the second paragraph of "Feature Calculations for Translation Efficiency") but we moved that discussion to this new paragraph in the main text. Finally, we added more covariates to Figure E7 (namely, all positions of the Kozac site and the average codon translation rate per gene). Although there are many other features that could be included in this figure, we do not feel like they have received widespread attention in literature. Their inclusion would also overlap previous studies and do not contribute to the main conclusions of our paper. As such, we feel that limiting ourselves to just this new set of covariates is sufficient.

8. *The sentence "However, we observe a correlation between position and slow outlier strength even when separating outliers by codon (Figure 5)" and Figure 5 itself are unclear.*

We have changed the sentence to "However, we observe a correlation between position and slow outlier strength even when conditioning on the codon (Figure 5), and thereby controlling for differences in codon usage at different positions within the gene". The intent is simply that we perform the same correlation but only on AAA codons, or AAC codons, etc. We have also modified the caption and title of Figure 5 to make this calculation more clear.

9. *Page 23, ribosome fragments -> ribosome-protected fragments*

We have made this change.

10. *In table E1, are these counts of tRNA genes, or tRNA molecules per cell, or some other unit?*

The units are number of reads per million (RPM). We have updated the table to reflect that.

11. *The meaning of figure E5 is unclear. for example, what are "fast outlier genes" and "position per length of ACA slow outliers" in the bottom right panel?*

We thank the reviewer for pointing out this confusion. To address this concern, as well as that of Reviewer 3, we first renamed "slow outlier genes" simply to "reduced TE genes" and "fast outlier genes" to "increased TE genes", in the main text, in the caption of Figure E6, and in Figure E6 (renumbered due to a new addition to the figure set). We believe this makes the notation less confusing (when thinking about this notation compared to slow outliers, which are positions as opposed to genes).

In addition, we added more description to the caption of Figure E6, which helps clarify the features being considered in each panel of the figure.

<u>Response to Reviewer #2:</u>

1. *Novelty - the question and the conclusion are not highly novel. Many recent studies, cited here, and beyond, have recently come to the same conclusion. The combination of tRNA deletion does represent some novelty.*

This paper contributes to the active debate about codon bias and translation efficiency, which has recently been discussed in prominent reviews, and which ties into robust analysis of ribosome profiling data.

We believe that experimental manipulation of tRNA abundance provides us the ability to test causal relationships that have remained unaddressed in numerous studies relying purely on correlative studies using endogenous tRNA levels and endogenous genes. This experiment qualitatively distinguishes this work from the other studies mentioned by the reviewer and enhances its impact

substantially.

We have expanded the “Discussion” (paragraph 1 and 2) to highlight the state of the prior literature, the controversial nature of the questions addressed by this study, and the ways in which these conflicts may be affected by data analysis. By performing more robust data analysis, as well as direct experiments, we are able to shed light on these highly-debated views and answer questions that could not be answered, but merely speculated on, in previous work.

2. *A conceptual point is still not in place: clearly many previous studies have shown a string correlation between the tAI or CAI and protein expression level. The current lack of correlation with TE is thus hard to explain. The author mention the paper by Kudla and Plotkin (which came to same conclusion as in the current work - that secondary structure, not codon optimality affect TE), who offer a compelling reasoning to the conundrum: highly expressed genes are evolutionarily pressured to be codon optimized more than lowly expressed genes. The current authors appear to support that notion. Yet, it is still hard to grasp, how could that evolutionary effect be relevant if codon optimization had no effect on TE. I'm very curious to read the authors' view on this, in a discussion.*

We thank the reviewer for highlighting this important and conceptually challenging point. We expanded the “Discussion” (paragraph 1) to elaborate further on this. Briefly, we do see that codon adaptation correlates with TE, but experimental manipulation of codon adaptation by changing cellular tRNA levels does not alter TE in the manner that would be predicted by a direct, causal effect of codon use on efficiency. We instead believe that codon usage bias arises from one or more selection forces. Since there is a positive correlation between TE and the per-gene average of elongation rates inferred by our model (a correlate added to Figure E7), and of smaller magnitude than that of tAI, one possible force is based on the view that ribosomes and translation factors are rate limiting for overall cellular growth, and so the ability to synthesize the cellular proteome (in which the translation machinery itself comprises a major fraction) with fewer ribosomes permits faster growth. Our data are also consistent with the general understanding that initiation is the rate-limiting step in translation, which is less controversial but which actually does entail that codon usage throughout the body of the gene does not affect its translational efficiency, but could impact the amount of ribosome*time required.

<u>Response to Reviewer #3:</u>

1. *Other merits of the paper include the use of DMS probing as opposed to computational prediction of mRNA structure -- something that should perhaps be mentioned in the abstract.*

We have added a phrase to the “Abstract” showing that mRNA secondary structure is calculated both computationally and from structure probing data.

2. *The authors should be more circumspect about the use of their regularization term when inferring dwell times. I suggest the authors perform an additional analysis using an expression that modulates the regularization term by a scaling factor, beta, and then show how their qualitative results vary with beta -- in order to determine to what extent this additional term, made for technical convenience, has influenced their inferred parameters and their conclusions.*

We appreciate this suggestion for a more careful analysis of our model. We performed the experiment as mentioned, whereby we vary the hyperparameter C, which controls the weight of the regularization term in the objective function. Throughout the paper, C is set to 100 (noted in paragraph 1 of the “Translation model” section of “Materials and Methods”). We appended to that same paragraph a description showing that the main results are agnostic to the value of C (namely, correlation between codon translation rate and codon bias, correlation to protein abundance, and outlier correlations). We also added Table E5 to summarize the results under these various models.

© European Molecular Biology Organization

3. *The word "yeast" should appear somewhere in the title or abstract, so that readers can quickly know what organism is being studied.*

We added “yeast” to the “Abstract”.

4. *For better or worse, the term TE has already been defined in the literature as simply RPF rpkm divided by mRNA rpkm. I agree that this is not always the best measure of actual synthesis rates per mRNA molecule, and I appreciate the attempt to rigorously estimate such rates from data using a mechanistic model. But nonetheless I encourage the authors to use a different terminology for their inferred synthesis rates, Jm/Mm -- as opposed to "TE", which already has a clear and consistent definition in the literature.*

We believe that translation efficiency rather represents the ribosome abundance compared to the mRNA abundance. We have seen this term used more generally in other papers to represent this quantity, regardless of how the numerator and denominator were calculated. We’d like to continue to use TE, but have made it clear at its first mention (in the “Introduction”) and elsewhere as appropriate that it is estimated TE from our model. Since we are trying to establish a rigorous technique for ribosome profiling data, similar to how mRNA abundance is extracted from RNA-seq data even with various model corrections, we’d like to try to keep the notation of TE, which is grounded in the biological phenomenon under study.

5. *I find Figure 5, and the entire notion of "outlier strength", difficult to interpret, despite several re-readings. Outlier strength is initially defined position by position, but it is then discussed in the context of "outlier genes". Is this simply an abuse of notation, or is different measure of "outlier" defined on a gene-by-gene basis, by summing over positions within each gene?*

We thank the reviewer for suggesting this clarification for the notation of outliers. As in our response to Reviewer 1, to address this concern, we first renamed “slow outlier genes” simply to “reduced TE genes” and “fast outlier genes” to “increased TE genes”, in the main text, in the caption of Figure E6, and in Figure E6 (renumbered due to a new addition to the figure set). We believe this makes the notation less confusing (when thinking about this notation compared to slow outliers, which are positions as opposed to genes).

In addition, we added more description to the caption of Figure E6, which helps clarify the features being considered in each panel of the figure. To specifically address the reviewer’s question, the features in Figure E6 were indeed calculated over all positions in the genes in the specified gene set (now either reduced TE or increased TE genes). For example, position per length for slow outliers is the distribution of k/L_m for all positions (m,k) such that (m,k) is a slow outlier.

6. *The authors could do a better job placing their results in the literature on determinants of protein synthesis. In particular, the authors write:*
"Although codon usage is a strong correlate to TE (Figure E6), our mutant experiments suggest (via the correlation between codon bias and tRNA abundance)…”
…I would suggest the authors re-write this part of their Discussion, to explain that their results support the widely held view on the direction of causality between codon bias and expression level -- a view that dates back to Andersson and Kurland (1990) and Bulmer (1991), and which has already been validated by manipulative experiments in microbes.
Likewise, the authors' statement that "our experimental results suggest a model in which initiation is rate-limiting in physiological conditions" is, again, completely in line with the standard view of translation dating back to Bulmer 1991.

This is an excellent point. We have added these references to the specific sections in the “Discussion” and in the “Introduction”, and have modified the text to show that our experiments directly support this model that has been previously suggested. In addition, we feel it is important to highlight that although this is a long-standing view, there have also been experiments showing the opposite, and the contrary view seems to be fairly commonly held.

2nd Editorial Decision 20 November 2014

Thank you again for submitting your work to Molecular Systems Biology. We have now heard back from the two referees who agreed to evaluate your manuscript. As you will see from the reports below, the referees are satisfied with the modifications made and they think that the study is now suitable for publication.

Before formally accepting your manuscript we would like to ask you to include the GEO accession numbers in the data availability section of your manuscript.

Thank you for submitting this paper to Molecular Systems Biology.

Reviewer #1:

The authors have removed my concerns about the manuscript by presenting additional experimental data and substantially revising the text.

Reviewer #3:

I have no further detailed comments; the authors have addressed my concerns and, it appears, the concerns of the other referees as well.

2nd Revision - authors' response 02 December 2014

Thank you for your final suggestions regarding our manuscript. The final version is enclosed.

The data used in this study is available using GEO series accession number GSE63789.

Please also note that in the duration, we had sequenced some samples at higher depth, and so felt that it would be useful to submit that version of the data to GEO. For consistency, we have updated the numbers and figures in the paper to match this data. No claims are changed -- many numbers were simply modifications in the second decimal place, and significant p-values values were generally made stronger.

© European Molecular Biology Organization
